# A systematic review and coordinate-based meta-analysis of resting-state fMRI in athletes from open and closed skills sports

**DOI:** 10.1038/s41598-025-07192-2

**Published:** 2025-07-01

**Authors:** Zhurui Yan, Mengqi Zhao, Yapeng Qi, Antao Chen, Hong Mou, Xize Jia, Yingying Wang

**Affiliations:** 1 School of Psychology, Shanghai University of Sport, Shanghai, China; 2 Center for Exercise and Brain Science, Shanghai University of Sport, Shanghai, China; 3 School of Psychology, Zhejiang Normal University, Jinhua, China; 4 Key Laboratory of Intelligent, Education Technology and Application of Zhejiang Province, Zhejiang Normal University, Jinhua, China

**Keywords:** Athletes, Resting-state functional magnetic resonance imaging, Functional connectivity, Activation likelihood estimation, Seed-based d mapping, Meta-analysis, Cognitive neuroscience, Motor control, Psychology

## Abstract

The impact of prolonged sports training on athletes’ brain functional activity remains inconclusive. A systematic review and coordinate-based meta-analysis of resting-state functional magnetic resonance imaging (rs-MRI) is necessary to identify functional connectivity changes induced by prolonged sports training. A total of 31 studies were included in the systematic review, and 18 studies with 347 non-athletes and 327 athletes were analyzed using coordinate-based activation likelihood estimation (ALE) and seed-based d mapping with permutation of subject images (SDM-PSI) meta-analysis. Results revealed that greater functional connectivity in athletes in the paracentral lobule, medial frontal gyrus, precuneus, inferior parietal lobule, supramarginal gyrus, inferior frontal gyrus, Rolandic operculum, and median cingulate/paracingulate gyri (DCG). Consistent changes in the DCG were identified in closed-skill sports athletes. Furthermore, increased regional functional activity was observed in the posterior cingulate gyrus, lingual gyrus and cerebellum. Both meta-analytical methods emphasize altered functional activity within the default mode network (DMN), cerebellar network (CBN), ventral attention network (VAN), visual network (VA), and sensorimotor network (SMN). These findings suggest that long-term sport training optimizes connection strength and efficiency in brain regions associated with visual attention, cognitive and motor control in athletes. Overall, our study reveals shared neural adaptations across different types of athletes, offering new insights into the effects of long-term specific training on brain functional connectivity in specialists.

**Supplementary Information:**

The online version contains supplementary material available at 10.1038/s41598-025-07192-2.

## Introduction

Long-term planned, structured, and cascaded professional training program not only significantly improves athletes’ behavioral performance and physical fitness^1–3^, but also fosters the development of complex cognitive processes, such as attention allocation, motor control, and decision-making, along with adaptive alterations in brain function and structure^4–8^. Notably, due to the distinct cognitive and motor demands across sport types, sports are commonly classified into open- and closed-skill disciplines^9^. In particular, open-skill sports (such as table tennis or football) require athletes to rapidly perceive and process cues in order to anticipate opponents’ movements and respond flexibly in unpredictable, externally paced, and dynamic environments. Training and brain functional adaptations in such sports are closely associated with heightened sensitivity to external stimuli, cognitive flexibility, inhibitory control, and rapid decision-making^10,11^. In contrast, closed-skill sports (such as swimming or track and field) are typically performed in stable, self-paced, and predictable environments, where athletes execute complex movements according to pre-established routines to achieve precise control over their internal states and optimize performance^12,13^. The underlying neural mechanisms in these sports may be more engaged in fine motor coordination and body control^14^. These domain-specific sport demands lead athletes to develop adaptive cognitive processing strategies and engage distinct neural mechanisms^10^.

Over the past decade, researchers have increasingly used functional magnetic resonance imaging (fMRI) to examine the neurobiological changes induced by prolonged sports training and the relationship between athletic expertise and brain function. Techniques such as resting-state fMRI (rs-fMRI) and task-based fMRI have been particularly effective in capturing changes in brain activity and connectivity across various motor disciplines, offering insights into how sports training shapes the neural mechanisms of athletes. Task-based fMRI studies have proven particularly valuable in revealing both shared and distinct patterns of brain activation across athletes from different sport types. On the one hand, recent meta-analyses have identified that athletes consistently exhibit greater activation in brain regions associated with sensorimotor processing, attention and memory, such as the inferior frontal gyrus (IFG), superior frontal gyrus (SFG), postcentral gyrus (PoCG), and the precentral gyrus (PreCG) during perceptual and motor response tasks^15,16^. On the other hand, these studies also highlight sport-specific functional adaptations. For instance, athletes from closed-skill sports, such as rowing and swimming, exhibited stronger activation in brain regions associated with internal state regulation and stable task performance, including occipitotemporal cortex (OTC) and posterior cingulate cortex (PCC), during working memory and action inhibition tasks^17^. In contrast, open-skill sports athletes, such as football players, demonstrated greater activation in brain regions related to working memory, cognitive control, and semantic information processing, such as cuneus, middle temporal gyrus (MTG), inferior parietal lobule (IPL), and angular gyrus, when performing visuospatial processing tasks^18,19^. These findings indicate that despite shared neural benefits from long-term sports training, distinct patterns of functional specialization emerge depending on the specific motor demands of each sport. Therefore, integrating research across both open-skill and closed-skill sports is essential for understanding how long-term athletic training drives functional brain plasticity in athletes, and for determining whether such sport-related neuroplasticity reflects common principles or unique adaptations across motor disciplines.

Rs-fMRI, which captures spontaneous neural activity and low-frequency fluctuations of the brain in the absence of tasks or external stimuli, has emerged as a powerful tool for evaluating resting-state functional connectivity (rs-FC) changes induced by motor learning or sports training in athletes^4,20^. Notably, altered rs-FC in athletes has been observed across a range of brain networks, like the salience network (SN), default mode network (DMN), frontoparietal network (FPN), ventral attention network (VAN), and sensorimotor network (SMN)^4,21–23^. Meanwhile, to characterize these alterations and the potential interconnection patterns across athletes types^24,25^, researchers have employed both seed-based FC and non-seed-based approaches, such as Region of Interest (ROI)-based FC, independent component analysis (ICA), (fractional) amplitude of low-frequency fluctuation (fALFF/ALFF), regional homogeneity (ReHo), voxel-mirrored homotopic connectivity (VMHC), degree centrality (DC) and graph theory analysis.

Specifically, seed-based FC is a widely adopted system-level approach for assessing brain connectivity in athletes, which calculates the correlation between the time series of a ROI and all other voxels in the brain to produce whole-brain connectivity maps, while ROI-based FC calculates the correlations between predefined ROI^26^. Studies using these approaches have identified altered FC in athletes in various brain regions, such as the cingulate gyrus (CG)^27–30^, PreCG^29–31^, PoCG^31–33^, IFG^22,33,34^, IPL^21,22,33,35^, precuneus^22,30^, MTG^34,36^, OTC^33,34,36,37^, and cerebellum (CB)^36,38^. Yet, considerable variability exists among athletes from different sports disciplines. For example, stronger FC between IPL and middle frontal gyrus (MFG) was found in basketball players^22^, while weaker FC between the parietal region and MFG was reported in badminton players^38^. Meanwhile, inter-sport comparisons have revealed that, compared to handball athletes, wrestlers showed increased FC in the parahippocampal gyrus, anterior orbitofrontal gyrus, and SFG, reflecting increased cognitive demands related to spatial navigation, memory encoding and sensorimotor integration^39^. Similarly, compared to competitive aerobics athletes, greater connectivity between the IFG and putamen was found in football players^40^, possibly reflecting enhanced responsiveness and inhibitory control related to their long-term specialized training^6,40,41^. Collectively, these findings suggests that differences in motor performance between athletes might be inseparably related to distinct patterns of rs-FC alterations.

In other non-seed-based approaches, ALFF/fALFF and ReHo are often combined to comprehensively capture the intensity and local synchronization of spontaneous brain activity^42,43^, but they detect different brain regions^20^. ALFF measures the amplitude of spontaneous fluctuations within a specific frequency band, while fALFF quantifies the proportion of low-frequency power relative to the entire frequency spectrum. ReHo quantifies local synchronization of neighboring voxels using Kendall’s coefficient of concordance. Related studies have found significant increased ALFF in MFG and MTG in ballroom dancing athletes^34^, while decreased ALFF in the MTG and weaker ReHo in the MFG in badminton and volleyball athletes^35,38^. ICA, a data-driven method, decomposes the brain’s voxel-wise time series into temporally and spatially independent components, allowing for the assessment of intra-network connectivity^44^. For example, Huang et al.^45^ observed that long-term intensive gymnastics training optimized both intra- and inter-network FC in regions associated with motor and executive functions, such as the DMN, CB, and FPN. Graph theory analysis has also been applied to examine the topological properties of brain networks, highlighting integration and segregation features. DC, one of the simplest and most computationally efficient graph metrics, reflects the importance of a node in information transfer^46^. Increased DC in the Calcarine (a visual area) has been found in both open- and closed-skill sports athletes^47^. VMHC, which evaluates inter-hemispheric connectivity, has revealed higher symmetry in bilateral PoCG, MTG, and superior temporal gyrus in elite basketball players^48,49^. Based on our current investigations, all these methods have their own advantages in studying sport-specific neural adaptations, but the neural correlates underlying differences in perceptual-cognitive capabilities and motor control process among athletes from different sports, potentially shaped by prolonged sport-specific training, have yielded inconsistent findings. It is necessary to analyze these metrics individually or in combination to better explore the extent to which they can detect athlete-specific changes in brain activity^50,51^.

Previous systematic reviews of rs-fMRI studies in motor specialists have highlighted that lengthy and extensive training promotes functional brain plasticity and that alterations may occur in special regions and network levels^4,52^. However, Calmels^52^ included heterogeneous samples (motor specialists with years of experience in skill motor activities, such as musicians), making it challenging to derive clear conclusions regarding the impact of long-term athletic training on changes in brain functional plasticity in athletes. Cantou et al.^4^ provided a detailed differentiation of previous studies in terms of methodology as well as type of expertise to determine whether there is a neural signature of domain-specific expertise, but a quantitative meta-analysis is still lacking. To address these gaps, the current meta-analysis builds on previous findings by applying rigorous screening and categorization protocols for athlete participants. By incorporating diverse rs-fMRI metrics, this research seeks to comprehensively identify the key brain regions and neural mechanisms associated with long-term motor training experience.

Furthermore, previous research has increasingly shown that athletes’ experience and training level (training intensity, duration, and other training-related variables) may be related to differences in cognitive function and brain activity^53,54^. Compared to amateur athletes or novices, elite athletes exhibit stronger FC and greater network efficiency in brain regions associated with sensorimotor, attentional regulation, and executive control, reflecting neural features related to advanced skill proficiency^49,55^. Therefore, to ensure meaningful differentiation of training-induced neural changes, this study focuses on athletes with at least three years of consistent training or competition experience, representing a relatively advanced stage of skill development. It is worth noting that sex differences may contribute to variability in cognitive functions and brain activity^56,57^, even though such differences may stem from genetic predispositions, hormonal influences, or lifestyle and sociocultural factors^39,58,59^. Nevertheless, our study primarily focuses on synthesizing previous findings to identify neuroplastic adaptations driven by long-term motor training experience, while also considering sex and training experience as potential sources of heterogeneity, with their moderating effect evaluated in subsequent analyses.

Here, in addition to conducting a systematic review of rs-fMRI on athletes with long-term training experience, this paper employs coordinate-based meta-analysis (CBMA), a quantitative approach that synthesizes voxel-based findings across multiple fMRI studies, to derive robust insights into resting-state brain functional activity^60^. Specifically, we integrate two widely used methods: activation likelihood estimation (ALE), which models the spatial convergence of activation probabilities, and seed-based d mapping with permutation of subject images (SDM-PSI), which preserves information about the direction and overall effect sizes. By combining these approaches, we achieve a more comprehensive and accurate characterization of the neural correlates of athletic expertise^50,51,61,62^.

Taken together, the present study has two primary objectives. First, we aim to identify consistent alterations in rs-FC across various indicators in athletes, reflecting experience-dependent brain plasticity. Second, we explore specific spatial convergence in brain activity between athletes from open- and closed-skill sports, provided the sample size is sufficiently large. Based on previous findings from task-based fMRI and rs-fMRI studies, we hypothesize that athletes would show brain functional changes in currently defined brain areas associated with perceptual-motor processing, attention, and cognitive control. Moreover, we expect different types of athletes would show distinct patterns of functional reorganization to meet the specific cognitive and motor demands of their respective sports^4,52,63^.

## Results

### Included studies and sample characteristics

**Fig. 1:**
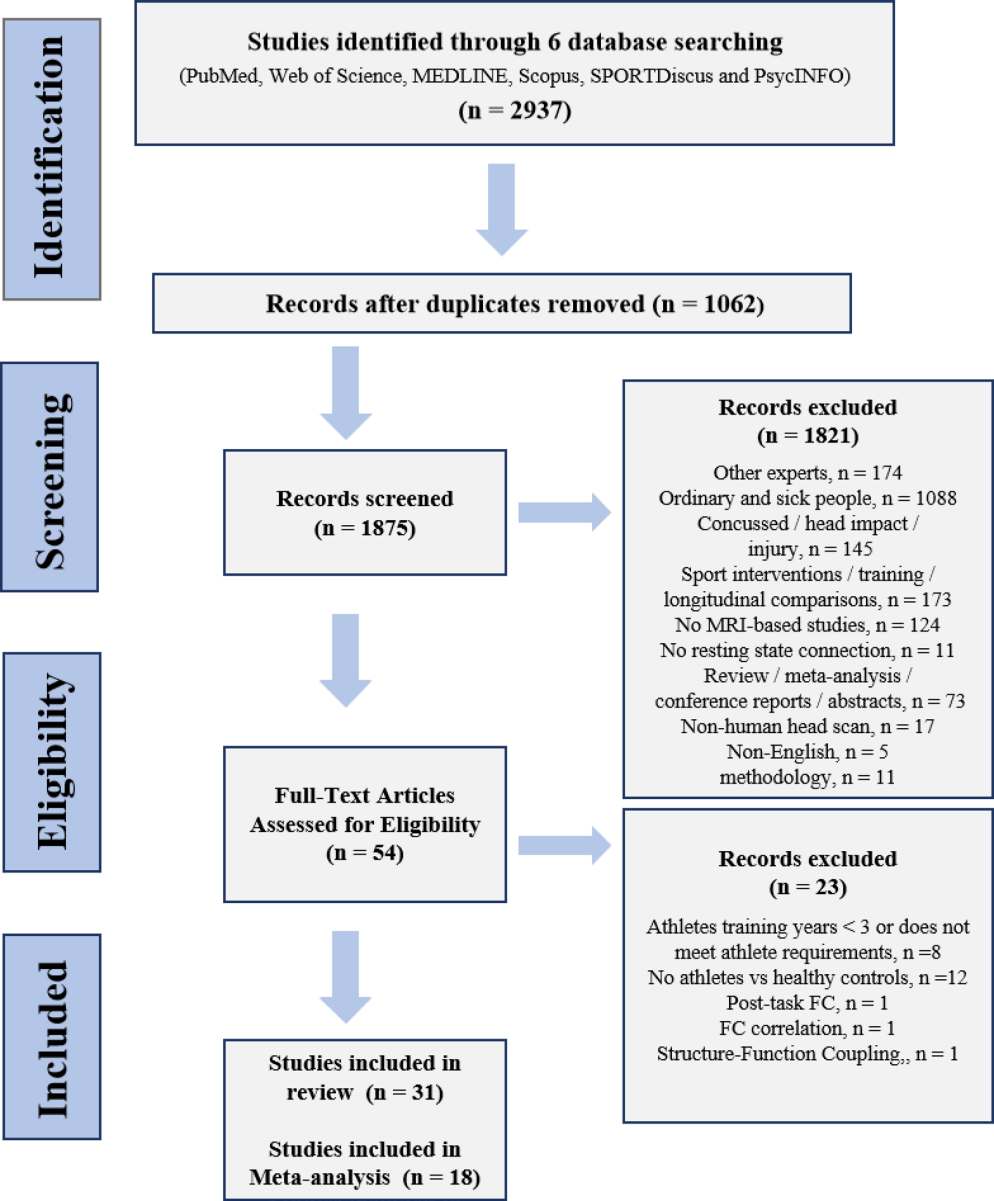
PRISMA flowchart of study selection. Abbreviations: FC: functional connectivity.

**Fig. 2:**
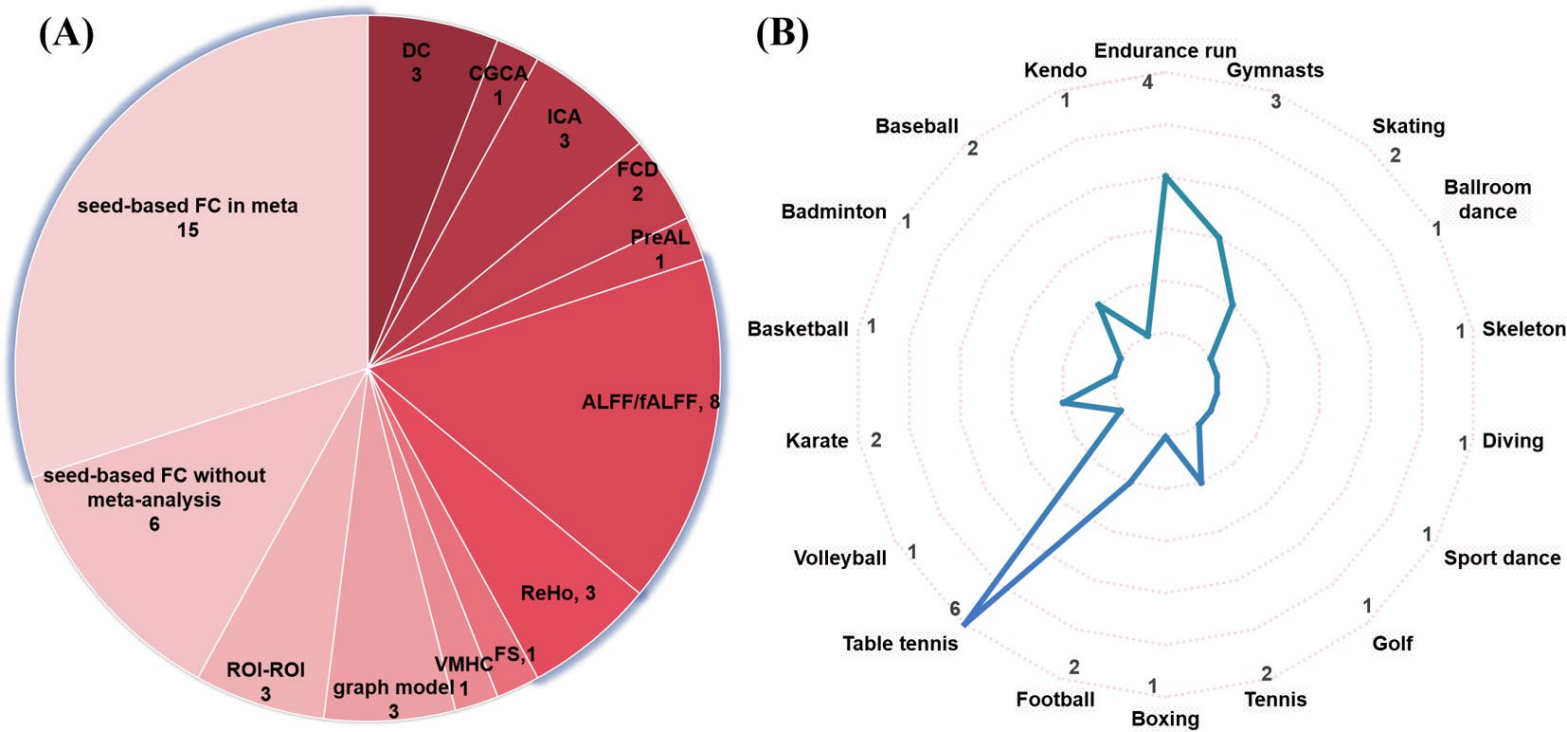
Methodological characteristics and sports program characteristics of the included studies. (**A**) A total of 21 seed-based FC (15 included in the meta-analysis) and 29 other non seed-based FC metrics (10 ALFF/fALFF/ReHo included in the meta-analysis) in the systematic review. (**B**) A total of 14 studies on closed-skill sports and 17 studies on open-skill sports were examined. Table tennis (n = 6), along with endurance running (n = 4) are the most researched. Abbreviations: ALFF: amplitude of low-frequency fluctuation; CGCA: conditional granger causality analysis; DC: degree centrality; fALFF: fractional amplitude of low-frequency fluctuation; FC: functional connectivity; FCD: functional connectivity density; FS: functional stability; ICA: independent component analysis; PreAL: percentage amplitude of fluctuation; ReHo: regional homogeneity; ROI: regions of interest; VMHC: voxel-mirrored homotopic connectivity.

A total of 2937 records were retrieved through systematic searches of PubMed, Web of Science, MEDLINE, Scopus, SPORTDiscus and PsycINFO databases (Fig. 1). After removing duplicate articles and further reading the titles and abstracts, we retained a total of 54 studies and excluded 1875 papers. After further screening the full text, we excluded a total of 23 studies that did not meet the inclusion criteria and discovered 31 papers satisfy the requirement in systematic review. Among them (Fig. 2A), 21 studies employed seed-based FC^21,22,27–29,31–38,64–71^, and 20 studies reported a total of 29 non-seed-based FC metrics, including ALFF/fALFF (n = 8)^34,35,37,38,40,66,68,72^, ReHo (n = 3)^28,35,68^; DC (n = 3)^40,68,72^, ICA (n = 3)^45,73,74^, Graph-based metrics (graph theory and network-based statistics) (n = 3)^28,63,65^, FCD (n = 2)^30,75^, ROI-based FC (n = 3)^40,64,76^, and other metrics, such as VMHC, CGCA, percentage amplitude of fluctuation (PerAF), and functional stability, each reported in one study^30,33,40,77^. Subsequently, we identified 18 studies eligible for the further meta-analysis, including seed-based FC (n = 15)^21,22,27–29,31,32,34–36,64–68^, regional functional activity metrics: ALFF/fALFF (n = 7)^34,35,38,40,66,68,72^ and ReHo (n = 3)^28,35,40,68^ (Table 1). Table 2 reports other studies that were not included in the meta-analysis. Supplementary Material 1 shows the articles included in the systematic review and their main findings. Supplementary Material 2 provides a detailed report of the articles excluded by full-text screening and the reasons for exclusion. Supplementary Material 3 outlines the fMRI data acquisition parameters (e.g., scanner type, field strength, sequence details) and preprocessing pipelines for each study included in the meta-analysis.

**Table 1:** Studies included in the meta-analysis (n = 18).

References	Sports	Skill types	Metrics	Athletes			Controls		Field	Score (12)
				N (M/F)	Age	TYs	N (M/F)	Age		
Jin et al.^66^	Skeleton	Closed	FC/ALFF	11(6/5)	22.9 (0.5)	4 (1.1)	23 (8/15)	21.2 (2.2)	3.0 T	11
Qi et al.^72^	Table tennis	Open	fALFF	25 (12/13)	20.0 (1.7)	11.9 (2.7)	33 (16/17)	20.8 (1.7)	3.0 T	10
Yan et al.^28^	Endurance run	Closed	FC/ ReHo	28(14/14)	22.5 (3.1)	8.5 (2.7)	31 (15/16)	21.4 (1.9)	3.0 T	11
Zhang et al.^35^	Volleyball	Open	FC/ALFF/fALFF/ReHo	10(0/10)	21.0 (1.3)	10.3 (1.5)	10 (0/10)	20.4 (0.7)	3.0 T	8
Zhou et al.^27^	Football	Open	FC	28(0/28)	–	9.9 (2.7)	29 (0/29)	–	3.0 T	9
de la Cruz et al.^65^	Endurance run	Closed	FC	17 (17/0)	28.8 (4.8)	≥ 3	21 (21/0)	26 (6.1)	3.0 T	9
Cao et al.^31^	Endurance run	Closed	FC	22 (22/0)	26.3 (6.1)	6.2 (2.4)	20 (20/0)	24.6 (4.1)	3.0 T	9
Ogino et al.^32^	Boxing	Open	FC	21 (21/0)	26.7 (4.0)	–	22 (22/0)	27.2 (3.8)	3.0 T	11
Zhang et al.^68^	Ice-skating	Closed	FC/fALFF /ReHo	19(–/–)	20.8 (1.9)	10.8 (3.9)	15 (–/–)	21.9 (2.6)	3.0 T	9
Shi et al. ^40^	Soccer	Open	ALFF	20 (0/20)	19.3 (1.4)	10.8 (2.1)	15 (0/15)	18.9 (1.0)	3.0 T	9
Berti et al.^64^	Karate	Open	FC	14 (8/6)	23.7 (4.1)	14 (6.8)	14 (10/4)	24 (2.9)	1.5 T	10
Lu et al. ^34^	Ballroom dance	Closed	FC/ALFF	23 (0/23)	20.8 (1.6)	9 (3.3)	21 (0/21)	20.8 (0.8)	3.0 T	11
Liu et al.^67^	Diving	Closed	FC	15(6/9)	13.3 (1.6)	> 4	15 (10/5)	14.3 (2.4)	3.0 T	11
Tan et al.^22^	Basketball	Open	FC	21 (21/0)	21.3 (1.3)	11.4 (2.3)	21 (21/0)	21.9 (0.8)	3.0 T	11
Kim et al.^29^	Sport dance	Closed	FC	12 (6/6)	21.9 (5.1)	6.8 (1.9)	12 (5/7)	22.1 (4.2)	3.0 T	10
Raichlen et al.^21^	Distance run	Closed	FC	11 (11/0)	21.6 (2.4)	–	11 (11/0)	20.6 (1.1)	3.0 T	10
Kim et al.^36^	Golf	Closed	FC	10 (0/10)	29 (2.3)	10 (2.1)	10 (0/10)	28.4 (3.1)	3.0 T	10
Di et al. ^38^	Badminton	Open	ALFF	20 (10/10)	22.5 (4.6)	8.9 (3.3)	18 (9/9)	20.7 (4.3)	3.0 T	10

ALFF, amplitude of low-frequency fluctuation; fALFF, fractional ALFF; FC, functional connectivity; N (M/F), number (male/female); ReHo, regional homogeneity; TYs, training years.

**Table 2:** Studies included in the systematic review but not included in the further meta-analysis (n = 24).

References	Sports	Skill types	Metrics	Athletes			Controls		Field
				N (M/F)	Age	TYs	N (M/F)	Age	
Studies using non-seed-based functional connectivity approaches (n = 16)									
Cao et al.^30^	Gymnasts	Closed	FCD, FS	13 (6/7)	20.5 (3.2)	16.1 (3.1)	14 (7/7)	20.3 (2.5)	3.0 T
Liu et al.^33^*	Table tennis	Open	PerAF	20 (12/8)	21.8 (2.9)	12.2 (2.8)	21 (12/9)	22.2 (2.5)	7.0 T
Qi et al.^72^	Table tennis	Open	DC	25 (12/13)	20.0 (1.7)	11.9 (2.7)	33 (16/17)	20.8 (1.7)	3.0 T
Yan et al.^28^	Endurance run	Closed	Graph theory	28 (14/14)	22.5 (3.1)	8.5 (2.7)	31 (15/16)	21.4 (1.9)	3.0 T
Zhang et al.^74^	Speed skaters	Closed	ICA	15 (15/0)	20.9 (1.8)	9.7 (3.6)	15 (15/0)	20.8 (1.9)	3.0 T
Li et al.^37^*	Table tennis	Open	sALFF, dALFF	20 (12/8)	22.2 (2.8)	7.1 (3.7)	21 (12/9)	22.6 (2.4)	7.0 T
de la Cruz et al. ^65^	Endurance run	Closed	NBS	17 (17/0)	28.8 (4.8)	≥ 3	21 (21/0)	26 (6.1)	3.0 T
Gao et al.^77^**	Little-ball	Open	CGCA	35 (17/18)	19.4 (3.7)	9.9 (0.4)	39 (24/15)	20.3 (1.3)	3.0 T
Zhang et al. ^68^	Ice-skating	Closed	DC	19 (–/–)	20.8 (1.9)	10.8 (3.9)	15 (–/–)	21.9 (2.6)	3.0 T
Gao et al.^75^**	Little-ball	Open	dFCD	33 (18/15)	19.6 (3.3)	10 (2.5)	36 (23/13)	20.1 (1.0)	3.0 T
Shi et al. ^40^	Soccer	Open	DC, VMHC,ROI-ROI	20 (0/20)	19.3 (1.4)	10.8 (2.1)	15 (0/15)	18.9 (1.0)	3.0 T
Berti et al.^64^	Karate	Open	ROI-ROI	14 (8/6)	23.7 (4.1)	14 (6.8)	14 (10/4)	24 (2.9)	1.5 T
Fujiwara et al.^76^	Kendo	Open	ROI-ROI	14 (14/0)	39.9 (13)	> 10	11 (11/0)	38.8 (13.2)	3.0 T
Duru and Balcioglu^73^	Karate	Open	ICA	13 (7/6)	22.3 (4.6)	> 10	13 (7/6)	26.7 (5.8)	3.0 T
Huang et al.^45^	Gymnasts	Closed	ICA	13 (6/7)	20.5 (3.2)	16.1 (3.3)	14 (7/7)	20.3 (2.5)	3.0 T
Wang et al.^63^	Gymnasts	Closed	Graph theory	13 (6/7)	20.5 (3.2)	> 12.5	14 (7/7)	20.3 (2.5)	3.0 T
Studies using seed-based functional connectivity approaches without further meta-analysis (n = 6)									
Liu et al.^33^*	Table tennis	Open	Static FC, dFC	20 (12/8)	21.8 (2.9)	12.2 (2.8)	21 (12/9)	22.2 (2.5)	7.0 T
Zheng et al.^71^*	Table tennis	Open	Static FC, dFC	20 (12/8)	21.8 (2.9)	12.2 (2.8)	21 (12/9)	22.3 (2.4)	7.0 T
Li et al.^37^*	Table tennis	Open	Static FC, dFC	20 (12/8)	22.2 (2.8)	7.1 (3.7)	21 (12/9)	22.6 (2.4)	7.0 T
Sie et al.^70^***	Baseball	Open	Seed-based FC	18 (18/0)	20.6 (1.7)	9.8 (2.7)	15 (15/0)	23 (1.9)	3.0 T
				15 (15/0)	23.3 (1.3)	4.2 (1.8)			
Chang et al.^69^***	Baseball	Open	Seed-based FC	18 (18/–)	20.5 (1.6)	9.3 (2.2)	17 (17/0)	23.1 (1.9)	3.0 T
				19 (19/–)	22.9 (1.5)	4.4 (2.6)			
Di et al.^38^ ****	Badminton	Open	Seed-based FC	20 (10/10)	22.5 (4.6)	8.9 (3.3)	18 (9/9)	20.7 (4.3)	3.0 T

ALFF, amplitude of low-frequency fluctuation; CGCA, conditioned granger causality analysis; dALFF, dynamic ALFF; DC, degree centrality; dFC: dynamic functional connectivity; dFCD: dynamic functional connectivity density; fALFF, fractional ALFF; FC, functional connectivity; FCD, functional connectivity density; FS, functional stability; ICA, independent component analysis; N (M/F), number (male/female); NBS, network-based statistics (a novel graph approach); PerAF, percentage amplitude of fluctuation; ROI, regions of interest; sALFF, static ALFF; TYs, training years; VMHC, voxel-mirrored homotopic connectivity.

*No further meta-analysis was performed due to large magnetic field differences. **Results were reported just for elite athletes who met the athletes’ requirements, and these studies focused on tennis and table tennis. ***Athletes included in the study were divided into two different skill level groups. ****The study used seed-based connectivity analysis with a predefined positive correlation mask.

#### Participants characteristics

Among the studies included in the systematic review, 14 studies focused on closed-skill sports and 17 studies on open-skill sports, encompassing a total of 18 distinct types of sports (Fig. 2B). Table tennis (n = 6), as a representative open-skill sport, along with endurance running (n = 4), as typical closed-skill sports, have been widely used as typical representatives to investigate the neural plasticity of athletes across different sport types. Among the studies included in the meta-analysis, while the participants in the studies conducted by Liu et al.^67^ were adolescents, they met our selection criteria for athletes and was not excluded. In addition, in the studies conducted by Ogino et al.^32^ and Raichlen et al.^21^, although the number of years of training for the athletes was not reported, the athletes were selected from professional committees or teams and actively participated in several sports competitions. The athletes in the other studies had more than 3 years of training and the number of athletes in each study ranged from 10 to 28. Moreover, 5 studies recruited only female athletes, 5 studies recruited male athletes, 1 study did not report on this, and the remaining studies included mixed-gender samples. Healthy control participants were matched for gender and age and did not have the corresponding specific skills training. The total population in our sample included 327 athletes and 341 healthy controls. These characteristics, including types of sports, demographic details of the participants (gender, number, age, and training years), as well as study quality scores, are detailed in Table 1.

#### Results of the quality and risk bias assessment of the studies

Each study included in the meta-analysis received a quality score higher than 8. The Kappa coefficient for agreement between the two researchers’ ratings was 0.70, which indicates that our assessment does not have a high risk of bias. Detailed evaluation scores can be found in Supplementary Material 4.

### Coordinate-based meta-analysis

#### Activation likelihood estimation meta-analysis

**Fig. 3:**
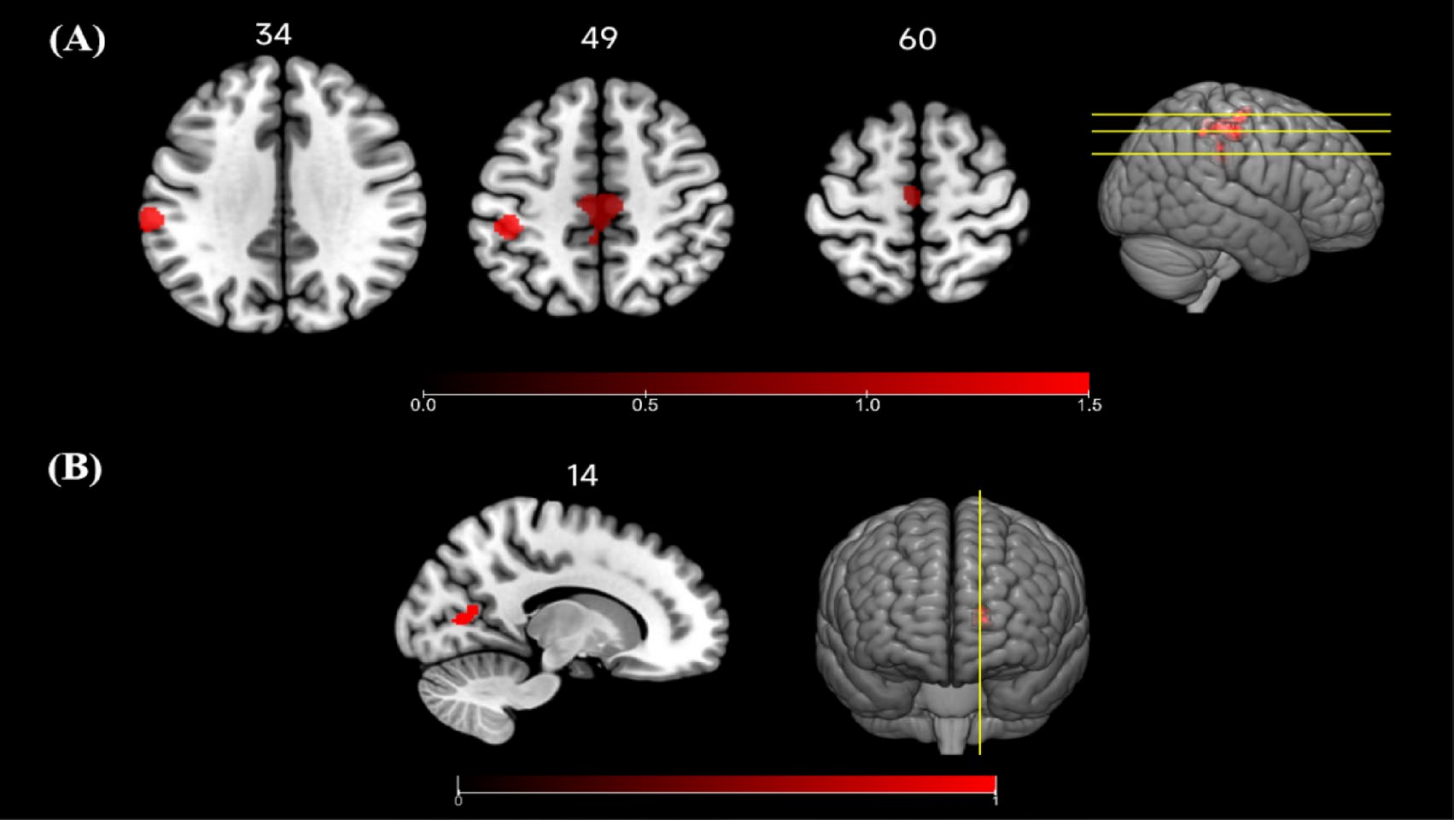
Results of altered resting-state functional activity in different types of athletes based on activation likelihood estimation meta-analysis. Compared with health controls, athletes showed (**A**) enhanced FC in PCL, MeFG, precuneus and IPL; (**B**) enhanced ALFF, fALFF, ReHo in right PCG and LG. Abbreviations: ALFF: amplitude of low-frequency fluctuation; fALFF: fractional ALFF; FC: functional connectivity; IPL: inferior parietal lobule; LG: lingual gyrus; MeFG: medial frontal gyrus; PCG: posterior cingulate gyrus; PCL: paracentral lobule; ReHo: regional homogeneity.

The seed-based rs-FC meta-analysis on 15 studies in both open- and closed-skill sports athletes was performed. The ALE meta-analysis results (14 experiments, 113 foci for athletes > non-athletes contrast) found that a significant cluster was located within a cluster of the 1568 mm^3^ centered on (4, − 22, 50), covering paracentral lobule (PCL) (78.6%), medial frontal gyrus (MeFG) (16.3%) and precuneus (5.1%). Another significant cluster, with a size of 928 mm^3^ and centered at (− 64, − 30, 34), covering IPL (100%), belonging to BA40 (Table 3; Fig. 3A).

**Table 3:** Activation likelihood estimation meta-analysis results in athletes.

	Peak clusters (BA)	MNI coordinates			z	Volume(mm^3^)	ALE value (10^−2^)	Jackknife sensitivity	Network	Cognitive function
		x	y	z						
FC										
1	PCL (BA 5, 31)	4	− 22	50	4.47	1568	1.48	12/14	SMN	Sensorimotor integration, lower-limb motor control
	MeFG (BA 6)							12/14	SMN	Motor preparation and initiation, high-level motor planning, attentional regulation, performance monitoring
	Precuneus (BA 7)							12/14	DMN	Self-awareness, episodic memory, spatial navigation
2	IPL (BA 40)	− 64	− 30	34	4.99	928	1.77	12/14	VAN	Motor understanding, hand–eye coordination, attentional reorienting, action observation and imitation
ALFF, fALFF, ReHo										
1	PCG (BA30)	16	− 64	14	3.92	608	0.94	7/10	DMN	Autobiographical memory, self-referential processing, contextual memory retrieval
	LG (BA 18)							7/10	VN	Visual information processing and recognition

ALE, activation likelihood estimation; ALFF, amplitude of low-frequency fluctuation; BA, brodmann areas; DMN, default mode network; fALFF, fractional ALFF; FC, functional connectivity; IPL, inferior parietal lobule; LG, lingual gyrus; MeFG, medial frontal gyrus; MNI, montreal neurological institute; PCG, posterior cingulate gyrus; PCL, paracentral lobule; ReHo, regional homogeneity; SMN, sensorimotor network; VAN, ventral attention network; VN, visual network.

In the subgroup meta-analysis, the results related to closed-skill sports athletes > non-athletes contrast (9 experiments, 63 foci) did not find significant results. No additional meta-analysis for athletes < non-athletes contrast was performed due to the insufficient number of experiments and coordinates (less than 5 experiments per contrasts).

The regional spontaneous activity meta-analysis on 9 studies in athletes was performed. The results (10 experiments, 51 foci for athletes > non-athletes contrast) found that a significant cluster was located within a cluster of the 608 mm^3^ centered on (16, − 64, 14), covering posterior cingulate gyrus (PCG) (87.5%) and lingual gyrus (LG) (12.5%), belonging to BA30 and BA 18 (Table 3; Fig. 3B).

#### Seed-based d mapping with permutation of subject images meta-analysis

**Fig. 4:**
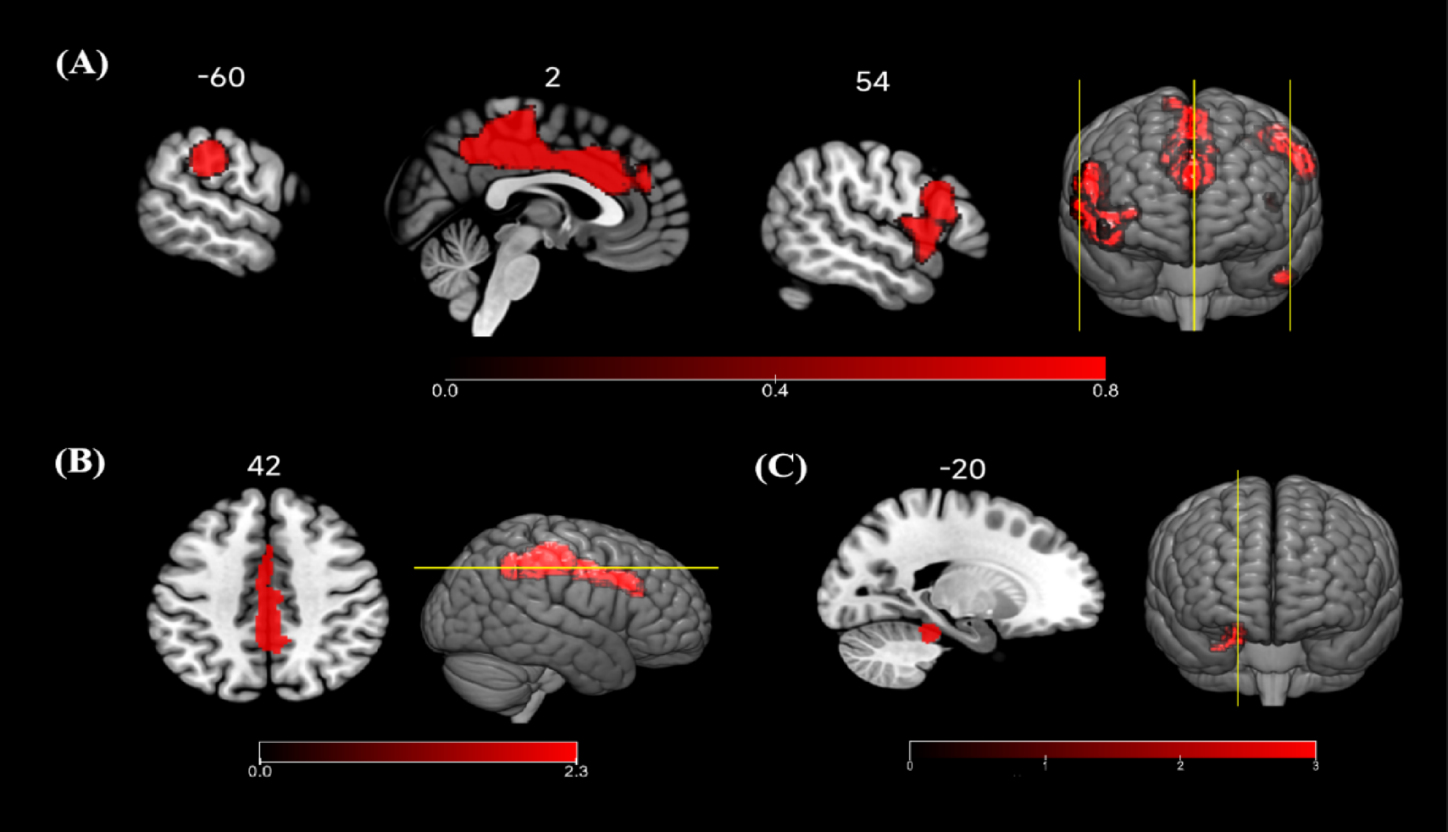
Results of altered resting-state functional activity in different types of athletes based on seed-based d mapping with permutation of subject Images meta-analysis. Compared with health controls, athletes showed (**A**) enhanced FC in DCG, SMG, RO and IFG; (**B**) enhanced FC in DCG in closed-skill sports athletes; (**C**) enhanced ALFF, fALFF, ReHo in CB. Abbreviations: ALFF: amplitude of low-frequency fluctuation; CB: cerebellum; DCG: median cingulate / paracingulate gyri; fALFF: fractional ALFF; FC: functional connectivity; IFG: inferior frontal gyrus; ReHo: regional homogeneity; RO: rolandic operculum; SMG:supramarginal gyrus.

The SDM-PSI meta-analysis results of seed-based rs-FC demonstrated four significant increased clusters in athletes. The first cluster was located in the right median cingulate and paracingulate gyri (DCG) (2, − 30, 50), with SDM-Z = 5.65, 95% CI [0.440, 0.908], *p* < 0.05, cluster size = 3235 voxels. The second cluster was located in the left supramarginal gyrus (SMG) (− 60, − 30, 36), with SDM-Z = 5.57, 95% CI [0.401, 0.837], *p* < 0.05, cluster size = 645 voxels, corresponding to BA 2. The third cluster was found in the right Rolandic Operculum (RO) (58, 6, 6), with SDM-Z = 4.57, 95% CI [0.315, 0.787], *p* < 0.05, cluster size = 346 voxels, corresponding to BA 48. The last cluster was located in the right IFG opercular (− 60, − 30, 36), with SDM-Z = 4.92, 95% CI [0.352, 0.818], *p* < 0.05, cluster size = 171 voxels, corresponding to BA 44 (Table 4; Fig. 4A). No significant clusters with decreased rs-FC were found in athletes compared with controls. In the subgroup meta-analysis, one significant cluster with increased rs-FC in closed-skill sports athletes was identified. This cluster was located in the left DCG (0, -36, 42), with SDM-Z = 4.49, 95% CI [0.331, 0.843], *p* < 0.05, cluster size = 2019 voxels (Table 4; Fig. 4B). No significant decreased region was found compared with controls. Overall, these results suggest that increased rs-FC in the DCG is consistently observed in closed-skill sports athletes.

**Table 4:** Seed-based d mapping with permutation of subject images meta-analysis results in athletes.

	Peak clusters (BA)	MNI coordinates			SDM-Z	Volume (mm^3^)	Effect size		Heterogeneity and publication bias		Jackknife sensitivity	Network	Cognitive function
		x	y	z			Hedges’ g	95% IC	*I* ^*2*^	Egger’s *p*			
FC													
1	DCG	2	− 30	50	5.65	3235	0.67	0.440, 0.908	5.28	0.537	15/15	DMN	Cognitive control, motor drive conflict and error monitoring
2	SMG, BA 2	− 60	− 30	36	5.57	645	0.62	0.401, 0.837	2.30	0.867	14/15	VAN	Action observation and imitation, language comprehension, bodily state perception, tool use
3	RO, BA 48	58	6	6	4.57	346	0.55	0.315, 0.787	6.06	0.416	12/15	CON SMN	Sensorimotor integration, bodily self-consciousness, speech production
4	IFG, BA 44	54	14	24	4.92	171	0.58	0.352, 0.818	2.28	0.840	13/15	VAN	Language production, motor inhibition, mirror neuron system, executive control, attentional regulation
Subgroup: closed-skill sports													
1	DCG	0	− 36	42	4.49	2019	0.59	0.331, 0.843	0.49	0.674	10/10		
ALFF, fALFF, ReHo													
1	CB, IV/V, BA 30	− 20	− 36	− 20	3.74	159	0.51	0.246, 0.782	4.41	0.278	6/10	CBN	Fine motor coordination, motor timing motor prediction, motor learning

ALFF, amplitude of low-frequency fluctuation; BA, brodmann areas; CB, cerebellum; CBN, cerebellar network; CON, cingulo-opercular network; DCG, median cingulate/paracingulate gyri; DMN, default mode network; fALFF, fractional ALFF; FC, functional connectivity; IFG, inferior frontal gyrus; MNI, montreal neurological institute; ReHo, regional homogeneity; RO, rolandic operculum; SMG, supramarginal gyrus; SMN, sensorimotor network; VAN, ventral attention network.

The SDM-PSI meta-analysis of regional spontaneous activity (ALFF, fALFF, ReHo) demonstrated a significant increased cluster in athletes. This cluster was found in the left CB, IV/V, with SDM-Z = 3.74, 95% CI [0.246, 0.782], *p* < 0.05, cluster size = 159 voxels, corresponding to BA 30 (Table 4; Fig. 4C). No decreased region was found compared with controls.

#### Jackknife sensitivity analysis and Heterogeneity analysis

The Jackknife sensitivity analysis for the ALE study found that the PCL, MeFG, precuneus and IPL were consistently identified in 12 out of 14 datasets, the PCG and LG were replicated in 7 out of 10 datasets. The Jackknife sensitivity analysis for the SDM-PSI study found that the DCG were replicated in 15 out of 15 datasets, the SMG were replicated in 14 out of 15 datasets, RO were identified in 12 out of 15 datasets, IFG were identified in 13 out of 15 datasets, and CB were replicated in 6 out of 10 datasets. These results validate the high reliability.

In addition, according to the I^2^ and Egger’s *p* (Table 4), no heterogeneity was detected in any significant clusters, and no evidence of publication bias was found. Details of the jackknife sensitivity analysis and funnel plots were given in Supplementary Material 5.

#### Meta regression analysis

No significant relationship was found between years of athletic experience, percentage of female athletes and seed-based FC or regional functional activity changes. Regression analyses based on MRI acquisition parameters (e.g., TR, TE) and preprocessing steps (e.g., spatial smoothing kernel size) are presented in Supplementary Material 5.

## Discussion

This systematic review and meta-analysis investigated resting-state brain functional activity of different types of athletes with lengthy-extensive specialized athletic training, compared with healthy non-athlete controls. The ALE meta-analysis revealed increased rs-FC in the PCL, MeFG, precuneus, and IPL, along with enhanced regional functional activity in PCG and LG in athletes. The SDM-PSI meta-analysis found altered rs-FC in DCG, SMG, RO and IFG in athletes, with consistent changes in DCG specifically observed in closed-skill sports athletes. Moreover, enhanced regional functional activity was observed in the CB. These results were supported by Jackknife sensitivity analysis. Definitely, the systematic review further analyzed the studies involving other rs-fMRI indicators, considering both local-level and network approaches across athletes engaged in different sports. The results show not only consistent functional alterations, but also specific changes in multiple brain regions and networks in both open- and closed-skill sports athletes. Overall, our findings are largely consistent with our hypotheses and previous meta-analysis on functional changes induced by exercise intervention^78^.

### Changes in seed-based functional connectivity in athletes

Seed-based rs-FC meta-analysis of athletes revealed that long-term participation in a variety of sports is associated with increased FC in precuneus and DCG, compared with health controls. Subgroup analysis also found consistent functional changes in DCG in athletes from closed-skill sports. As core nodes of the DMN, these regions play pivotal roles in environmental monitoring, situational memory and theory of mind^4,79^. Specifically, the DCG, an important component of the CG^80^, is crucial for cognitive control, conflict detection, as well as sensorimotor functions^81^. The precuneus is involved in spatial information processing and dynamically tracks and updates self-centered goals in dynamic environments^82^. Studies from both open- and closed-skill sports athletes have reported enhanced FC between the DMN and CB SMN, and temporal regions^21,27,28^. This reinforced FC is thought to directly correlate with their exceptional somatosensory processing abilities and may contribute to superior action perception, refined action selection, and efficient goal-directed motor planning capabilities^83^. In all, these neural reorganization enable athletes to effectively integrate proprioception with multimodal sensory feedback, ultimately allowing for the precise organization and coordination of body movements in accordance with external environmental demands and internal motor requirements.

In addition, long-term sports training also induces specific plasticity in higher-order motor control networks, evidenced by increased FC in the PCL, MeFG, and RO. The RO, located near the central sulcus^84^, serves as a key node in both the cingulo-opercular network (CON) and SMN^85–88^. It is involved not only in speech production, but also in regulating bodily self-awareness by integrating proprioceptive and interoceptive signals. The PCL, located at the frontoparietal junction, above the CG and below the precuneus, is a medial extension of the PreCG and PoCG^89,90^. The MeFG, situated along the medial wall of the prefrontal cortex (PFC) (containing supplementary motor area (SMA), pre-SMA, the frontal eye fields and dorsomedial PFC), governs critical functions including motor sequence encoding, spatial attention modulation, and behavior monitoring^91,92^. These regions are central to sensorimotor integration and motor planning^89^. Cross-sport studies involving skeleton athletes and soccer players have revealed a remarkably consistent pattern of FC enhancement^27,66^. This neural connectivity optimization across sports suggests that different disciplines may drive similar cognitive-motor pathways through distinct neural adaptations. These adaptations facilitate the efficient transformation of motor strategies from perception to execution and action anticipation^93^. Typical examples include the neural activation advantages and improved prediction abilities exhibited by badminton and table tennis athletes in direction anticipation and deceptive action recognition tasks^94,95^. In summary, these findings collectively indicate that elite athletic performance relies not only on precise sensory-spatial representations but also on the ability to interpret opponents’ movement intentions in dynamic environments, ultimately achieving action selection and modulation^96^. These results align with a previous systematic review that long-term skilled motor activities may be closely associated with functional plasticity in motor-related brain regions^4^.

The study further revealed enhanced FC in the left IPL, SMG, and right IFG, which are core nodes of the VAN, particularly supporting stimulus-driven attentional reorientation and the interpretation of observed actions. The right IFG, located in BA44^97^, plays a critical role in action inhibition, execution, attentional control, and spatial processing. It is also involved in language production and, as a major component of the mirror neuron system, participates in action observation and understanding^98–100^. The IPL serves as a crucial neural basis in the integration of sensory-motor information and motor execution, playing a key role in action understanding^101^. Increased FC between the IPL and PoCG in basketball players and boxers supports the cognitive role of IPL in action execution and observation^22,32^. The SMG, a lateral core subregion of the IPL, not only mediates body state perception and spatial orientation^102^, but also integrates higher-order cognitive functions such as complex tool use^103^, action imitation/recognition, and language comprehension^104^. Enhanced FC between the left SMG, right IFG, RO and calcarine cortex (a visual cortex) observed in volleyball and endurance athletes may reflect long-term sport-specific optimization of visual attention and multimodal sensory-motor integration. This adaptation likely provides neural support for top-down control of visual motion processing and real-time decision-making, facilitating the suppression of distracting signals, precise spatial localization, and the optimization of neural representations of action intentions in rapidly evolving competitive scenarios^28,35^.

### Changes in regional brain functional activity in athletes

We observed altered regional functional activity in the LG, CB and PCG. The PCG, located at the posterior part of the CG, is the core posterior hub of the DMN and plays a crucial role in spatial orientation, memory, and self-referential cognition^105–107^. Additionally, it is involved in decision-making processes when faced with risky and uncertain choices^106^. Enhanced local functional activity in the PCG may support the rapid processing of visuospatial information and facilitate decision-making in dynamic and unpredictable movement contexts^28^. As a crucial component of the ventral OTC^108,109^, the LG not only participates in primary visual information processing, but also integrates dynamic visual signals for target tracking and prediction^110^. The higher ReHo in the left LG of endurance athletes confirms that athletes’ superior athletic performance is inseparable from their ability to recognize visual information in environments over time^28,66^. The CB VI/V, located in the anterior mid-vermis and lateral lobules, are core nodes for motor regulation and support real-time prediction of movement parameters (including spatial positioning, directional adjustment, and speed integration)^111,112^. Earlier studies have confirmed a co-activation pattern between the SMN and CB IV-V during simple finger-tapping tasks^113,114^. This predictive motor mechanism shows specific enhancement in expert athletes. For instance, football and basketball experts demonstrate increased local functional activity in the bilateral anterior CB^38,115^, suggesting that long-term specialized training may induce neuroplastic changes of the cerebellar motor prediction network.

### Potential mechanisms of long-term athletic training

Our coordinate-based meta-analysis reveals the effects of long-term motor training on resting-state functional activity in specific brain regions of athletes. Prolonged motor training is associated with enhanced functional activity, particularly in regions linked to sensorimotor and attentional abilities^52^. From this, we believe that after a certain period of sports training, enhanced functional activity in brain regions associated with higher cognitive functions, particularly those related to attention, cognitive control and self-monitoring, may reflect the continuous optimization of integrative and attentional regulation processes during motor training. This strengthened connectivity is often driven by the repeated co-activation of these relevant brain regions, highlighting the potential impact of motor training on the coordination of neural networks^116^. In other words, motor execution and the processing of external information require the continuous engagement of relevant brain regions during motor training, leading to enhanced functional connectivity to adapt to the demands of the sports. Thus, functional plasticity of these areas may be directly shaped by long-term sports training.

Other studies included in the systematic review corroborated findings consistent with the meta-analysis. For instance, increased FCD in the VN was reported in open-skill sports players^75^, increased dFC in the VN, DMN and SMN was observed in table tennis players and gymnasts under 7T scanning^30,33^. Additionally, stronger FC in the SMN, EVN and DMN of elite closed-skill sport athletes was identified using ICA approach^63,74^. These convergent results suggest that prolonged sport-specific training induces neuroplastic adaptations in motor and attentional control networks, enhancing both motor stability and cognitive efficiency in complex environmental demands. Furthermore, at the level of functional network topological properties, diverse reorganizations across multiple networks were found in athletes^77^. In open-skill sports, such as football, the CBN showed significantly elevated node efficiency and more efficient information transmission, supporting dynamic integration of multisource information. Conversely, the SMN displayed adaptive reductions in nodal efficiency, reflecting a “deductive streamlining” of the motor execution network^115^. Closed-skill sport athletes (e.g., endurance running) exhibited a “resource-conserving” topological adaptation: the CBN sacrificed broad integration capacity to strengthen selected critical pathways to meet relatively single task requirements, while the SMN nodal efficiency enhancements optimized motor control^28^. Additionally, the observed reductions in network flexibility and modular cohesiveness among gymnasts may reflect a neural specialization and automation of motor skills characteristic of elite-level training^117^.

Both local-level and network approaches have enhanced our understanding of athletic neuroplasticity. Long-term athletic training may be related to sport-specific reorganization of functional connectivity between brain regions and networks among athletes participating in different types of sports. Comprehensive testing of these metrics can lead to a deeper understanding of brain plasticity from prolonged activity. However, as encountered during our data selection process, the heterogeneity in sports classification, participants characteristics (e.g., age, gender composition, years of training, level of expertise and cultural background, genetic predispositions and the degree of cognitive engagement), and control group composition (e.g., level of non-sport physical activity and lifestyle) may confound brain functional connectivity outcomes and should not be overlooked. Meanwhile, the resting-state BOLD signals are influenced by cardiovascular health parameters, such as heart rate and blood pressure^118^, and lower heart rates have been linked to enhanced connectivity in central autonomic and sensorimotor networks in endurance athletes^65^. Therefore, to improve the reliability and interpretability of future findings, studies should adopt stricter recruitment criteria, explicitly analyze the influence of gender, and systematically assess and control for physiological confounders.

### Limitations and future directions

While the coordinate-based meta-analysis demonstrated relative reliability through sensitivity analyses and systematic review validation, several limitations must be acknowledged to guide future research.

First, technical variability in fMRI acquisition (e.g., scanner type, field strength, and sequences) and preprocessing pipelines (e.g., motion correction, physiological noise regression, smoothing kernels size, thresholding, and reporting standards) across included studies may introduce non-neural variance, thus limiting the comparability of results. Although I^2^ statistics assessed heterogeneity in effect sizes, they cannot account for these methodological differences. Clear reporting of data quality control is needed to improve replicability.

Second, in seed-based FC analysis, the selection of seed regions (e.g., definitions, coordinates, network assignments) exhibits inherent subjectivity across studies, which impacts the characterization of connectivity patterns. The limited numbers of studies not only hinders efforts to classify seeds by their corresponding functional networks for subgroup analysis, but also prevents the inclusion of other metrics (e.g., ICA, graph theory) that could synthesis capture additional network adaptations. Similarly, small sample sizes hinder subgroup analysis for open-skill sports, preventing validation of common neural or program-specific functional alterations. Future meta-analyses should incorporate diverse analytical approaches and larger, more representative samples to better revisit connectivity patterns within specific functional networks and capture underlying neural mechanisms.

Meanwhile, this meta-analysis relied on coordinate-based methods rather than original statistical maps, which may exclude widespread but subthreshold activations. Additionally, overly dispersed coordinates across studies can reduce the stability of meta-analytic outcomes. Future work should advocate for open sharing of statistical maps to enable image-based meta-analyses (IBMA) for higher precision^119^. Emerging analytical approaches, such as machine learning, provide new insights into understanding the bridging relationship between resting-state FC and task functions^120^. Continuous refinement of keyword sets and the expansion of search strategies, combined with the integration of coordinate-based and data-driven methods, will improve literature retrieval and enrich systematic reviews of motor cognitive neural mechanisms. Furthermore, since sports training can induce functional and structure changes^121^, exploring the relationship between functional and structural connectivity in response to sports training has become an increasingly important focal point.

Publication bias is another potential limitation of the current meta-analysis, although Egger’s test did not reveal significant publication bias. We cannot fully exclude the possibility that studies with non-significant or null results remain unpublished. Future meta-analyses should include unpublished or pre-registered studies to enhance result robustness.

## Conclusions

In all, the results of our review and coordinate-based meta-analysis indicate that long-term sports training is associated with shared alterations in resting-state functional activity among athletes, regardless of sports type. These alterations are mainly characterized by enhanced connectivity efficiency in brain regions related to visuospatial attention, cognitive control and sensorimotor processing. Meanwhile, athletes participating in open- or closed-skill sports also showed specific connectivity patterns, possibly reflecting adaptations to the cognitive demands of their respective training programs. These findings support the association between long-term sports training and brain functional plasticity. However, it is important to acknowledge that the observed neural differences may also be influenced by other factors, such as genetic predisposition, cognitive engagement, or non-sport physical activity. Therefore, while this meta-analysis provides supporting evidence for functional plasticity in athletes, further empirical research is needed to more precisely elucidate how different long-term training patterns shape neural structure and functional adaptations.

## Methods

### Study design

This systematic review and meta-analysis of resting-state brain functional changes in athletes followed the Preferred Reporting Items for Systematic Reviews and Meta-Analysis (PRISMA) guidelines (http://www.prisma-statement.org)^122^ and previously published high-quality meta-analysis of neuroimaging studies^61,62,123^. The protocol was registered in the Prospective Register of Systematic Reviews (CRD42022383164). Since our study is a meta-analysis of previously published studies and does not involve human participants, ethical approval and participants consent are not applicable.

### Search strategy

We conducted a systematic search of the PubMed, Web of Science, MEDLINE, Scopus, SPORTDiscus and PsycINFO databases to reduce the risk of missing relevant studies, covering relevant studies published from the initial emergence of resting-state research around 2010 until 2024^124^. During the screening process, the following three categories of keywords were searched: (1) terms related to the field of athletes or sports (‘athletes’ or ‘players’ or ‘sports’ or ‘experts’ or ‘profess*’ or ‘training’ ) AND (2) terms related to magnetic resonance imaging (‘functional magnetic resonance imaging’ or ‘resting-state functional magnetic resonance imaging’ or ‘resting-state’ or ‘rs-fMRI’) AND (3) terms related to functional connectivity indicators (‘connectivity’ or ‘functional connectivity (FC)’ or ‘rs-FC’ or ‘amplitude of low-frequency fluctuation (ALFF)’ or ‘regional homogeneity (ReHo)’ or ‘degree centrality (DC)’ or ‘independent component analysis (ICA)’ or ‘voxel-mirrored homotopic connectivity (VMHC)’). Following initial screening, we expanded our analysis to include additional metrics reported in the retrieved literature, such as graph theory, functional connectivity density (FCD), granger causality analysis (GCA) and dynamic FC (dFC). The references of the included studies and relevant review articles were manually checked to provide additional studies. The authors updated the results of the search every five months using the same search strategy to include additional eligible studies, with the most recent update conducted on November 13, 2024. Detailed descriptions of search strategies and processes are provided in Supplementary Material 6.

### Study exclusion and inclusion criteria

After excluding duplicate articles retrieved from the different databases, we excluded articles based on their title and abstract according to the following criteria: (1) participants enrolled in the article were non-athletes (animals, patients, musicians or other motor skill training specialists); (2) studies of athletes with a history of head impact or current brain injury or sports-related concussion; (3) studies that performed training interventions or longitudinal comparisons; (4) studies using non-MRI neuroimaging techniques; (5) studies that did not assess resting-state brain functional network connectivity; (6) systematic reviews or meta-analysis; (7) conference reports or abstracts; and (8) studies not in English. In addition, we also excluded studies that used machine learning methods because these were ROI-based and emphasized predictive model performance, rendering them incompatible with ALE analysis and hypotheses^125^.

The articles were included in the system review if they met the following criteria: (1) compared healthy athletes and non-athletes (healthy general participants); (2) had at least 10 participants in each group^61^; (3) included athletes have at least 3 years consistently training in a single, specific sport to improve his sport performance or athletic achievement (> beginner level) or actively participated in official national/regional competitions as an individual or team member^126,127^; (4) results were presented using standard Talairach or Montreal Neurological Institute (MNI) coordinates.

Considering the number of studies required and the statistical power in ALE and SDM meta-analyses, we established the following inclusion criteria, focusing exclusively on whole-brain analyses. The fALFF/ALFF and ReHo indices, which reflect patterns of local functional activity, were analyzed collectively. For studies comparing athletes with different skill levels, we excluded those that did not perform direct group comparisons between experts and novices using two-sample t-tests. Additionally, rs-fMRI data obtained using 7T scanners were not combined with lower-field datasets, due to they exhibit substantially higher proportions of physiological noise and require more advanced motion correction, denoising, and distortion correction than 1.5T/3T scanners^128^. For studies using 1.5T/3T scanners, although head motion and other physiological confounds (e.g., physiological noise) are known to influence rs-fMRI results, these variables were not used as explicit inclusion criteria due to insufficient and inconsistent reporting across the included studies.

### Identification of papers and extraction of data

Two experimenters independently extracted the basic information from the articles using a standard table, including the publication information, type of sports, characteristics of participants (gender, age, training duration, sample size), neuroimaging acquisition and preprocessing parameters (MRI field, scanning parameters, motion correction methods, statistical thresholds), and main results. For studies with incomplete methodological reporting, supplementary materials were thoroughly examined. Additionally, they also extracted the peak coordinates of brain regions with significant differences between groups and recorded the reasons for inclusion or exclusion. When coordinate data were missing or inconsistent, corresponding authors were contacted for clarification. Disagreements regarding article identification or extraction were resolved through discussions with a third investigator.

### Risk of bias and methodological quality assessment

Two experimenters individually assessed the rigour for each article (participants and methodological quality) using a 12-point checklist based on previously published structural MRI and rs-MRI meta-analysis^127,129^. Subsequently, they individually used a modified version of the Newcastle–Ottawa Scale (NOS), which is specific to fMRI data, to assess the risk of bias^130,131^. Inter-rater agreement coefficients were calculated to quantify the level of agreement, and scores were categorized as follows: 0–3 (high risk of bias), 4–7 (moderate risk of bias), and 8–11 (low risk of bias). Any disagreement between raters will be discussed with the third investigator who will make the final decision. Detailed scoring information is provided in Supplementary Material 5.

### Coordinate-based meta-analysis

Since previous studies recommend including at least 8–10 studies when doing ALE or SDM-PSI meta-analysis^132,133^, the following coordinate-based meta-analysis were performed for indicators of seed-based FC and regional functional activity metrics (ALFF/fALFF/ReHo).

#### Activation likelihood estimation meta-analysis

ALE is a brain neuroimaging meta-analytic technique that treats loci of activation reported in fMRI studies as three-dimensional Gaussian probability distribution centers. Using established algorithms and spatial modeling, ALE creates an activation map and verifies convergence of the significant differences across studies^134–136^.

We used GingerALE 3.0.2 software (http://brainmap.org/ale) to explore the spatial convergence of brain hypoconnectivity or hyperconnectivity in athletes across the included studies. The “Talairach to SPM” conversion function in GingerALE was used to convert the Talairach spatial coordinates to MNI spatial coordinates. Based on previous comparisons of spatial specificity and sensitivity for different correction methods^25,137^ and selected thresholds from published meta-analysis^51,61,138^, we performed the group-contrasts analysis using cluster-level family-wise error with a cluster-level threshold at *p* < 0.05, voxel-level threshold at *p* < 0.001, and 5000 threshold permutations. The results were visualized with the MRIcroGL software (https://www.mccauslandcenter.sc.edu/mricrogl/).

In the specific meta-analysis, we conducted one contrast for ALFF/fALFF/ReHo: athletes > control group and two contrasts for seed-based FC: athletes > control group and closed-skill sport athletes > control group. At the same time, we attempted to classify the seed regions from the included studies based on the brain network model proposed by Yeo et al.^139^ to identify potential connectivity patterns within specific brain networks^125,140^. However, due to the limited sample size, we did not further analyze brain network-based changes or conduct subgroup analysis for open-skill sports athletes or contrast of athletes < control group.

#### Seed-based d mapping with permutation of subject images meta-analysis

Unlike ALE meta-analysis, SDM-PSI meta-analysis uses a random-effects model and threshold-free cluster enhancement statistics, integrating the reported activation coordinates and t-values reported in fMRI, to convert peak data into whole-brain effect size maps. By iteratively optimizing the effect size, the SDM is able to better estimate the direction (positive or negative) of the activation and the strength of the effect^141^.

We used SDM-PSI (version 6.23, https://www.sdmproject.com) to explore critical brain regions in athletes. Based on the coordinates extracted based on ALE, t-values corresponding to each coordinate were collected. For studies reporting only z-values or p-values, we converted them into t-values using SDM online converter (http://www.sdmproject.com/utilities/?show=Statistics). During preprocessing, we estimated the lower and upper bounds of the most probable effect size images. Subsequently, a random-effects model was employed together with the meta-analysis of non-statistically significant unreported effects (MetaNSUE) approach to estimate Hedges’ g and its standard error (SE) for each voxel. The 95% confidence interval (CI) was then calculated accordingly^141,142^. In accordance with SDM-PSI guidelines and in published meta-analysis^60,143,144^, we processed the data using the following default parameters: the default kernel size and thresholds: full anisotropy = 1, isotropic full width at half maximum (FWHM) = 20 mm, voxel size = 2 mm; the significance report: FWE correction method (1000 permutations, *p* < 0.05, cluster extent ≥ 10 voxels) with threshold-free cluster enhancement (TFCE).

In the specific meta-analysis, we conducted meta-analyses on studies focusing on both seed-based FC and local functional activity indices. Subgroup analyses of open- and closed-skill sports athletes were not performed.

### Jackknife sensitivity analysis, heterogeneity and publication bias

To check the robustness and reliability of the findings, we conducted the Jackknife sensitivity analyses. We removed data from one study at a time and performed a similar ALE or SDM-PSI analysis using GingerALE or SDM-PSI for the remaining study data set^51^. This procedure was repeated for each study. We deemed the results highly reliable and sensitive if they were observed in more than 50% of the studies^137,145^. Heterogeneity of results and publication bias were further explored using SDM-PSI. Effect sizes were estimated by extracting the I^2^ statistics and the Egger’s test *p*-value. Results were considered to exhibit low heterogeneity and no evidence of publication bias when I^2^ < 50%, Egger’s *p* > 0.05^146,147^.

### Meta regression analysis

To explore the potential influence of demographic variables (such as years of training, mean age, and gender ratio) as well as MRI acquisition and preprocessing procedures on the observed differences across indices, we performed meta-regression analyses. An FWE threshold of *p* < 0.05 was applied to determine statistical significance^148^.

## Electronic supplementary material

Below is the link to the electronic supplementary material.

Supplementary Material 1.

## Data Availability

The data underlying the findings of this study are available from the supplementary material or corresponding author on reasonable request.
