# Supplementary material for: A systematic review and coordinate-based meta-analysis of resting-state fMRI in athletes from open and closed skills sports
Source: Sci Rep. 2025 Jul 1;15:21870. doi: 10.1038/s41598-025-07192-2 (PMC12214797; doi:10.1038/s41598-025-07192-2)
Supplement: Supplementary file 1 — Supplementary Material 1. [file 41598_2025_7192_MOESM1_ESM.docx]

**Supplementary Material**

**A systematic review and coordinate-based meta-analysis of resting-state fMRI in athletes from open-skill and closed-skill sports**

Zhurui Yan^1,2^, Mengqi Zhao^3,4^, Yapeng Qi^1,2^, Antao Chen^1,2^, Hong Mou^1,2^, Xize Jia^3,4*^, Yingying Wang^1,2*^

*^1^ School of Psychology, Shanghai University of Sport, Shanghai, China.*

*^2^ Center for Exercise and Brain Science, Shanghai University of Sport, Shanghai, China*

*^3^ School of Psychology, Zhejiang Normal University, Jinhua, China*

*^4^ Key Laboratory of Intelligent, Education Technology and Application of Zhejiang Province, Zhejiang Normal University, Jinhua, China*

Zhurui Yan and Mengqi Zhao have contributed equally to this work.

**Corresponding Author:**

^*^Yingying Wang

No.399 Changhai Road, Yangpu District, Shanghai, China, 200438;

Tel: +(86)02165507525; Fax: +(86)02165507525;

Email: [wangyingying@sus.edu.cn](mailto:wangyingying@sus.edu.cn)

^*^Xize Jia

No.688 Yingbin Road, Jinhua, Zhejiang Province, China, 321004；

Tel:18969130297

Email: jiaxize@foxmail.com

**Supplementary Material 1**

**Table 1. Articles included in the systematic review and their main findings.**

| **Studies** | **Sports** | **Indices** | **Findings** |
| --- | --- | --- | --- |
| **Studies using non-seed-based functional connectivity approaches (n = 20, indices = 29).** | | | |
| Cao et al., 2024 | Gymnasts | FCD  FS | **A>H**: L/R AG; L/R ITP in Global/long-range FCD, FS;  **A<H:** R Pre in FS and Global /long-range /short-range FCD. |
| Jin et al., 2024 | Skeleton | ALFF | **A>H**: L fus; L ITG; L/R IFG; L MTG; IN, L RO; L STG |
| Liu et al., 2024 | Table tennis | PerAF | **A<H:** R STG; R Pre (0.01–0.08 Hz); L SFG; L LG; R PreCG; L SMA (0.198–0.25 Hz); R LG; L PCL; R SMA (0.073–0.198 Hz) ; R ITG; L put; R Pre; R PreCG (0.027–0.073 Hz) ; R MTG; R SFG; L MOG; L PoCG; R SFG (0.01–0.027 Hz) |
| Qi et al., 2024 | Table tennis | DC | **A>H**: vmPFC; **A<H**: L CS |
|  |  | fALFF | **A>H**: L IFG |
| Yan et al., 2024 | Endurance run | ReHo | **A>H**: R Cal; R PC; L/R LG; R PreCG; R PoCG; L/R Cun; **A<H**: L Cere_9; R Cere_crus2. |
|  |  | Graph theory | **A>H**: Nodal efficiency and local efficiency in motor cortex area; |
|  |  |  | **A<H**: Nodal efficiency and local efficiency in Cere. |
| Zhang, J., et al., 2024 | Volleyball | m/zALFF  m/zfALFF | **A>H**: R Para; L/R Cal; L ITG; L Cere;  **A<H:** R MFG; R SFG. |
|  |  | ReHo | **A>H**: R Cal (z, Sm KCCReHo); **A<H**: R MFG; R SFG (z, Sm KCCReHo);  **A>H**: R Cun; L/R Cal (z, Sm Cohe ReHo). |
| Zhang, K., et al., 2024 | speed skaters | ICA | 1. **H:** R AG; L Pre (DAN); L fus; R M1(FPN); L SMA(SMN); R PVC(VIS);   **A<H**: STG(DAN); R PFC(FPN) |
| Li et al., 2023 | Table tennis | sALFF | **A<H**: L ITG |
| Gao et al.,2023* | Little-ball | CGC | **EA>H**: R IFG to R SOG; **EA<H**: R Pre to R IFG. |
| de la Cruz et al., 2022* | Endurance run | NBS | **A>H**: Network component ^#^1 (13 nodes and 13 edges); ^#^2 (11 nodes and 13 edges). |
| Zhang et al., 2021 | Ice-skating | fALFF | **A>H**: R Cere |
|  |  | ReHo, DC | **A>H**: L Cere |
| Gao et al.,2021* | Little-ball | dFCD | **EA>H**: R LG |
| Shi et al., 2020 | Soccer | DC | **A>H:** L MFG; SMA, pall; L/R MFG; cau; put; tha; |
|  |  | ALFF | **A>H**: L SMA; L/R cau; put; tha; |
|  |  | VMHC | **A>H**: L/R SFG; IFG; SMA; cau; put; pall; GPe; tha; |
|  |  | ROI-ROI | **Closed-skill sports A>H:** L/R IFG; Cau and put;  **Open-skill sports A>H:** R IFG, L/R put; GPi and GPe; |
| Berti et al., 2019 | Karate | ROI-ROI | **A>H**: L pall and R STG; L MTG and R Hip |
| Fujiwara et al., 2019 | Kendo | ROI-ROI | **A<H**: R NA and R FEF. |
| Lu et al., 2018 | Ballroom dance | ALFF | **A>H:** L MTG; L/R PreCG; L/R IFG; L PoCG; L ITG; R MOG; R STG; L MFG; **A<H:** L LG. |
| Duru & Balcioglu., 2018 | Karate | ICA | **A>H:** Cun; MFG; STG (DMN); R PreCG; R SFG; L IFG; R Cere. |
| Huang et al., 2018 | Gymnasts | ICA | **A>H**: EVN; **A<H:** BG; aDMN; L/R FPN; SMN-BG; CB-PVN; BG-PVN; DMN-FPN. |
| Wang et al., 2015 | Gymnasts | Graph theory | **A<H**: local/global efficiency. nodal strength and efficiency, FC in Cere, FPN, CON. |
| Di et al., 2012 | Badminton | ALFF | **A>H:** L/R Cere; **A<H**: L SPL; L Pre; L MTG. |
| **Studies using seed-based functional connectivity approaches without further meta-analysis (n=6).** | | | |
| Liu et al., 2024 | Table tennis | static FC  dynamic FC | **A<H**: R SFG (R RO; SMG; L PoCG); R PreCG (L/R RO; L PoCG; PreCG); L MOG (L IFG; R SMG); R ITG (R cere; MFG; Pre; L SMA); L PoCG (L MTG; IFG; IPL; R MFG, AG); L LG (R Pre); R Pre (R Cal); R LG (L IOG); L SFG (R SFG).  **A>H**: R ITG (L/R cere; R Cal; PreCG; L Cun; MOG); L PoCG (R cere; Cal; L IFG; CG); L LG (L/R IOG;L STG; MOG; IOG; R Cal; IFG; SPG); R Pre (R Cere; Cal; MTG; MFG; SFG; L AG; SFG; Cun; STG;); R LG (R IOG; CG; L fus; SFG; IN; MOG; IFG; SFG); L SFG (L LG; AG; R SFG; AG; IPG). |
| Zheng et al., 2024 | Table tennis | dynamic FC | **A>H**: BA 11 (R Hip; R LG; R Cere VI; L RG); BA 48 (R Cere; L IOG).  **A<H**: BA 11 (R ORBsup; R Cere; L LG; L Cere VIII; L SMG; L IN); BA 48 (R SFG; L PoCG). |
| Li et al., 2023 | Table tennis | static FC | **A<H**: L ITG (L IPG; R MTG) |
|  |  | dynamic FC | **A<H**: L ITG (L IPG; L MFG; L SFG; L mSFG) |
| Sie et al., 2019 | Baseball | Seed-based FC | **A>H**: L amyg (R put); R pMCC (L/R PoCG; L/R IPL; L/R SMG); R aIN (R dACC);  **A<H**: R aIN (R PreCG); L amyg (L IPL; L PoCG); |
| Chang et al., 2018 | Baseball | Seed-based FC | **A>H:** R MTP (L Pre) |
| Di et al., 2012 | Badminton | Seed-based FC | 1. **H**: R Cere (L dlPFC; L PC; L TL; L/R SMA); L SPL (L/R dlPFC; L/R PC; L/R MT lobe; L/R Cere); L PC (R ACC; L/R MFG; R CG);   **A<H**: R Cere (R ACC); L PC (L IFG; L/F MFG) |
| **Included studies using seed-based functional connectivity approaches in the meta-analysis (n=15).** | | | |
| Jin et al., 2024 | Skeleton | Seed-based FC | **A>H**: SFG (L put; amgy; R Pre; CG; R SFG; PCL); IN (R IFG; MTG; STG; R AG; Pre; SMG). |
| Yan et al., 2024 | Endurance run | Seed-based FC | **A>H**: R Cal (L/R CG; R IFG); L Cere_9 (L/R CG; R IFG; L Pre); R Cere crus2 (L/R CG). |
| Zhou et al., 2024 | Football | Seed-based FC | **A>H**: foot execution region (R MTG); aMTG (R ACC; L MFG; L/R PCL; R SFG); aITG (L CG; R PCL); aTFC (L SPL; L prec; L SMG; L IPL; L PoCG); **A<H:** TP (R STG; R MTG); aPHG (R MTG). |
| Zhang, J., et al., 2024 | Volleyball | Seed-based FC | **A>H**: Cal (L PreCG; L SMA; R_IN; R RO; L SMG; L IPL). |
| de la Cruz et al., 2022** | Endurance run | Seed-based FC | **A>H**: L aIN (L PMC; R IN; R dlPFC; L/R vACC; L AG); R aIN (L IN; L/R vACC; L SMG); dACC (L/R M1; L/R vACC; L AG; R PMC). |
| Ogino et al., 2021 | Boxing | Seed-based FC | **A>H:** L OFC (L Hip; R MTG); L Put (R IFG; L M1; L PoCG). |
| Zhang et al., 2021 | Ice-skating | Seed-based FC | **A>H**: L Cere (L Cere; R Fus). |
| Cao et al., 2020 | Endurance run | Seed-based FC | **A>H**: L PreCG (R PreCG; R PoCG); L Hip (L SMA; L MCC); R Hip (L Cere). |
| Berti et al., 2019 | Karate | Seed-based FC | **A>H:** L pall (R STG; R MTG); R Hip (L CG; L MTG; L SMG; L MTG (R OCC; L/R Hip);  **A<H:** L pall (L/ R OCC); STG (L/R OCC); R Hip (R SFG); L MTG (R SFG; R Cere). |
| Lu et al., 2018** | Ballroom dance | Seed-based FC | **A<H**: L IFG (L/R IN; R ITG; L/R PreCG; L MTG; L Fus; R Cere). |
| Tan et al., 2017 | Basketball | Seed-based FC | **A>H:** R pre (L/R IFG; R MFG); L IN (L STP; R MFG; R IFG); R ACC (L SFG); L IFG (L IPL); L IPL (L MFG). |
| Liu et al., 2017 | Diving | Seed-based FC | **A>H**: L/R SFG (L ITG; R Pons); L/R IFG (L ITG; R VPC); L/R IN (L Pons; R LG; L Cere; L/R Hip (L Pons; L ITG); L/R Cere (L Cere; L ITG);  **A<H**: L/R SFG (R Cau); L/R IFG (Put); L/R IN (R Put); L/R Hip (R Cere; L MB); L/R Cere (L LL). |
| Raichlen et al., 2016 | Distance run | Seed-based FC | **A>H**: L aPFC (R SFG; R SMA); R IPL (R SFG; R MFG); L IPL (L PCL);  **A<H**: mPFC (R PoCG); CG (R PreCG; R SPL); L IPL (L/R OCC); R MC (L CG). |
| Kim et al., 2016 | Sport dance | Seed-based FC | **A>H:** PreCG (R CG; R OFG; R IFG; R MFG, L IFG, R PoCG; R sub-gyral). |
| Kim et al., 2015 | Golf | Seed-based FC | **A>H**: L Cere (L Cun; L MFG; L ITG; L STG; R MTG; R CG; R PC). |

Note: * Results were reported just for elite athletes who met the athletes’ requirements, ** The seeds reported are appeared in the article. Abbreviations: A: athletes; ACC: anterior cingulate cortex; aDMN/pDMN: anterior and posterior default mode networks; AG: angular gyrus; aIN, anterior insular cortex; aITG: anterior inferior temporal gyrus; ALFF: amplitude of low-frequency fluctuation; aMTG: anterior middle temporal gyrus; amyg: amygdala; Ang: angular; aPFC: anterior prefrontal cortex; aPHG: anterior parahippocampal gyrus; aTFC: anterior temporal fusiform cortex; BA: brodmann area; BG: basal ganglia network; Cal: calcarine; Cau: caudate; Cere/CB: cerebellum; CG: cingulate gyrus; CGC: conditioned granger causality; CON: cingulo-opercular network; CS: calcarine sulcus; Cun: cuneus; dACC: dorsal ACC; dALFF: dynamic ALFF; DAN: dorsal attention network; DC: degree centrality; dFCD: dynamic functional connectivity density; dlPFC: dorsal-lateral prefrontal cortex; EA: elite athletes; EVN: extrastriate visual network; fALFF: fractional amplitude of low-frequency fluctuation; FC: functional connectivity; FCD: functional connectivity density; FEF: frontal eye field within the motivation network; FG: frontal gyrus; FPN: fronto-parietal networks; FS: Functional stability; fus: fusiform gyrus; GPi: internal globus pallidus; GPe: external globus pallidus; H: health control group; Hip: hippocampus; ICA: independent component analysis; IFG: inferior frontal gyrus; IN: insula; IOG: inferior occipital gyrus; IPL: Inferior parietal lobule; IPG: inferior parietal gyrus; ITG: inferior temporal gyrus; L: left; LG: lingual gyrus; m: mean; LL: limbic lobe; M1: primary sensorimotor cortex; MB: Midbrain; MC: motor cortex; MCC: middle cingulate cortex; MFG: middle frontal gyrus; MOG: middle occipital gyrus; mPFC: medial PFC; mSFG: medial SFG; MTG: middle temporal gyrus; MTP: middle temporal pole; NA: nucleus accumbens; NBS: network-based statistics (a novel graph approach); OCC: occipital cortex; OFC: orbitofrontal cortex; OFG: occipital fusiform gyrus; ORBsup: orbital frontal superior gyrus; pall: pallidum; Para: paraHippocampal; PC: posterior cingulate; PCL: paracentral Lobule; PerAF: percentage ALFF; PFC: prefrontal cortex; PMC: premotor cortex; pMCC: posterior midcingulate cortex; PoCG: postcentral gyrus; Pre: Precuneus; PreCG: precentral gyrus; put: putamen; PVC: primary visual cortex; PVN: primary visual network; R: right; ReHo: regional homogeneity; RG: rectus gyrus; RO: Rolandic Operculum; ROI: regions of interest; SA: sensorimotor areas; sALFF: static ALFF; SFG: superior frontal gyrus; SMA: supplementary motor area; ReHo; SMN: sensorimotor network; SMG: supramarginal gyrus; SOG: superior occipital gyrus; SPL: Superior Parietal Lobule; STG: superior temporal gyrus; STP: superior temporal pole; tha: thalamus; TL: temporal lobe; TP: temporal pole; vACC: ventral ACC; vmPFC: ventromedial prefrontal cortex; VMHC: voxel-mirrored homotopic connectivity; VIS: visual network; VPC: visual primary and association cortex; z: z-transformed.

**Supplementary Material 2**

**Table 2. Articles excluded and exclusion reasons (n=23).**

| **Studies** | **Sports** | **Skill Types**  **/Athletes** | **Indices** | **Field** | **Analysis or Reasons for exclusion** | **Reasons for exclusion detailed exclusion reasons** |
| --- | --- | --- | --- | --- | --- | --- |
|  |  |  |  |  |  |  |
| Abbasi et al.,2024 | open | Football | Seed-based FC, DC | 3T | Subjects were football athletes only. | No healthy control group |
| Fitzgerald et al.,2024 | open | Football | FC self‑similarity | 3T | Compare FC of football and non-collision athletes in pre- and post-season. | No healthy control group |
| Li et al., 2024 | open | Football | Graph theory | 3T | Novice athletes with training of 2.35 ± 0.87. | No healthy control group |
| Peng et al., 2024 | open  closed | Soccer, Gymnasts, Swimmers, Volleyball, Basketball, Martial arts, Track and field. | DC | 3T | Included athletes of different sports types | Not fulfilling the condition of specific sport |
| Zuidema et al., 2024 | open | Football | Seed-based FC;  ALFF; ReHo | 3T | Control group were noncontact sports team | No healthy control group |
| DiFabio et al., 2023 | open | Ice hockey | ROI-ROI | 3T | Compared FC of FPN-DMN in pre- and post-season. | No healthy control group |
| Wu et al., 2023 | closed | Ballroom dance | Seed-based FC | 3T | FC Correlation analysis. | Focus on correlation of FC and the score |
| Zhou et al., 2023 | open | Tennis, table tennis | CGC | 3T | Years of training less than two years; cerebellar functional segregation and integration. | Not fulfilling the condition of training years |
| Li et al., 2022 | open | Combat | DC; ALFF; FC | 3T | Adolescent athletes with an average of 34.1 months of training and less than 3 years. | Not fulfilling the condition of training years |
| Monroe et al., 2022 | open | Roller derby | Structure-Function Coupling | 3T | structural coupling and decoupling. | Not just focus on rs-FC |
| Roberts et al., 2022 | open | Female varsity | ICA | 3T | Subjects were female varsity athletes only. | No healthy control group |
| Wilson et al., 2022 | open | Contact sports | ICA | 3T | Subjects were collision contact sports athletes. | No healthy control group |
| Zhang et al., 2022 | closed | Aerobic, anaerobic | ALFF, DC | 3T | Compared Sprinters with runners. | No healthy control group |
| Kim & Han, 2021 | open  closed | Sports dancers, golf, Taekwondo players | FC, fALFF | 3T | Included athletes of different sports types | Not fulfilling the condition of specific sport |
| Li et al., 2021 | open | Combat | ICA | 3T | Adolescent athletes with an average of 34.1 months of training and less than 3 years. | Not fulfilling the condition of training years |
| Manning et al., 2020 | open | Rugby | ICA | 3T | Compare rugby players, swimmers and rowers. | No healthy control group |
| Yang et al., 2020 | open | Fast-Ball Sports | gFCD | 3T | Novice athletes with an average of 1.98 years of training and less than 3 years. | Not fulfilling the condition of training years |
| Zhuang et al., 2020 | open | Fighters | Static / dynamic FC | 3T | Compare between healthy subjects and fighters and (2) between non-impaired and impaired fighters. | No compare between healthy control and non-impaired fighters |
| Gao et al., 2019 | open | Little-ball | SFC | 3T | Optimal connectivity distance. | Not fulfilling the condition of training years |
| Burzynska et al., 2017 | closed | Dancing | ROI-ROI | 3T | FC of the Motor Learning Network and AON.  Involves different dance genres | Not fulfilling the condition of specific sport |
| Churchill et al., 2017 | open | Varsity | Gconn | 3T | Compare non-contact, contact, and collision sports athletes. | No healthy control group |
| Huang et al., 2017 | closed | Swimming | DC; ROI-ROI;  Seed-based FC | 3T | Subjects were Olympic, elite and high swimming athletes. | No healthy control group |
| Muraskin et al., 2016 | open | Baseball | Seed-based FC | 3T | Seed-based post-task FC of L/R SMA. | Not the same as non-task-related rs-FC |

Note: Abbreviations: AON: Action Observation Network; ALFF: amplitude of low-frequency fluctuation; CGC: conditional Granger causality; DC: degree centrality; DMN: default mode networks; FC: functional connectivity; FPN: fronto-parietal networks; ICA: independent component analysis; Gconn: Global functional connectivity; gFCD: global functional connectivity density; SFC: stepwise functional connectivity; ReHo: regional homogeneity; ROI: region of interest; rs-FC: resting-state FC.

**Reference**

Abbasi, P., Fallahi, A., Nourshahi, M., Asadi, Y., Soltanian-Zadeh, H., & Nazem-Zadeh, M.-R. (2024). The impact of brain functional connectivity on skill and physical performance in soccer players: A resting state fMRI study. *Psychol Sport Exerc*, *76*, 102741. <https://doi.org/10.1016/j.psychsport.2024.102741>

Burzynska, A. Z., Finc, K., Taylor, B. K., Knecht, A. M., & Kramer, A. F. (2017). The Dancing Brain: Structural and Functional Signatures of Expert Dance Training. *Frontiers in human neuroscience*, *11*, 566. doi:10.3389/fnhum.2017.00566

Churchill, N. W., Hutchison, M. G., Di Battista, A. P., Graham, S. J., & Schweizer, T. A. (2017). Structural, Functional, and Metabolic Brain Markers Differentiate Collision versus Contact and Non-Contact Athletes. *Frontiers in neurology*, *8*, 390. doi:10.3389/fneur.2017.00390

DiFabio, M. S., Smith, D. R., Breedlove, K. M., Pohlig, R. T., Buckley, T. A., & Johnson, C. L. (2023). Altered brain functional connectivity in the frontoparietal network following an ice hockey season. *European Journal of Sport Science, 23*(5), 684-692.

Fitzgerald, B., Bari, S., Vike, N., Lee, T. A., Lycke, R. J., Auger, J. D., . . . Talavage, T. M. (2024). Longitudinal changes in resting state fMRI brain self-similarity of asymptomatic high school American football athletes. *Sci Rep, 14*(1), 1747. doi:10.1038/s41598-024-51688-2

Gao, Q., Yu, Y., Su, X., Tao, Z., Zhang, M., Wang, Y., . . . Chen, H. (2019). Adaptation of brain functional stream architecture in athletes with fast demands of sensorimotor integration. *Hum Brain Mapp, 40*(2), 420-431. doi:10.1002/hbm.24382

Huang, Z., Davis, H. I. V., Wolff, A., & Northoff, G. (2017). Thalamo-sensorimotor functional connectivity correlates with world ranking of Olympic, elite, and high performance athletes. *Neural plasticity, 2017*. doi:10.1155/2017/1473783

Kim, S. M., & Han, D. H. (2021). Comparison of brain activity within the sensorimotor network between sports players and patients with somatic symptom disorder. *Journal of Psychosomatic Research*, *147*, 110534. https://doi.org/10.1016/j.jpsychores.2021.110534

Li, J., Cao, Y., Huang, M., Li, Z., Qin, Z., & Lang, J. (2024). The alterations of functional brain networks and its relationship with sport decision-making and training duration in soccer players across different skill levels. *Neuroscience Letters*, *831*, 137788. [doi:10.1016/j.neulet.2024.137788](https://doi.org/10.1016/j.neulet.2024.137788)

Li, W., Kong, X., & Ma, J. (2022). Effects of combat sports on cerebellar function in adolescents: a resting-state fMRI study. *Br J Radiol, 95*(1130), 20210826. doi:10.1259/bjr.20210826

Li, W., Kong, X., Zhanng, Y., Luo, Y., & Ma, J. (2021). Effects of combat sports on functional network connectivity in adolescents. *Neuroradiology, 63*(11), 1863-1871. doi:10.1007/s00234-021-02713-y

Manning, K. Y., Brooks, J. S., Dickey, J. P., Harriss, A., Fischer, L., Jevremovic, T., . . . Menon, R. S. (2020). Longitudinal changes of brain microstructure and function in nonconcussed female rugby players. *Neurology, 95*(4), e402-e412. doi:10.1212/wnl.0000000000009821

Monroe, D. C., DuBois, S. L., Rhea, C. K., & Duffy, D. M. (2022). Age-Related Trajectories of Brain Structure-Function Coupling in Female Roller Derby Athletes. *Brain sciences, 12*(1). doi:10.3390/brainsci12010022

Muraskin, J., Dodhia, S., Lieberman, G., Garcia, J. O., Verstynen, T., Vettel, J. M., Sherwin, J., & Sajda, P. (2016). Brain dynamics of post‐task resting state are influenced by expertise: Insights from baseball players. *Human Brain Mapping*, *37*(12), 4454-4471. doi:10.1002/hbm.23321

Peng, Z., Xu, L., Wang, H., Song, T., Shao, Y., Liu, Q., & Weng, X. (2022). The lateralization of spatial cognition in table tennis players: neuroplasticity in the dominant hemisphere. *Brain Sciences*, *12*(12), 1607. https://doi.org/10.3390/brainsci12121607

Roberts, S. D., Wilson, A., Rahimi, A., Gorbet, D., Sergio, L., Stevens, W. D., & Wojtowicz, M. (2022). Investigation of baseline attention, executive control, and performance variability in female varsity athletes. *Brain Imaging Behav, 16*(4), 1636-1645. doi:10.1007/s11682-022-00635-8

Wilson, A., Stevens, W. D., Sergio, L., & Wojtowicz, M. (2022). Altered Brain Functional Connectivity in Female Athletes Over the Course of a Season of Collision or Contact Sports. *Neurotrauma reports, 3*(1), 377-387. doi:10.1089/neur.2022.0010

Wu, X., Lu, X., Zhang, H., Wang, X., Kong, Y., & Hu, L. (2023). The association between ballroom dance training and empathic concern: Behavioral and brain evidence. *Hum Brain Mapp, 44*(2), 315-326. doi:10.1002/hbm.26042

Yang, C., Luo, N., Liang, M., Zhou, S., Yu, Q., Zhang, J., . . . Gao, Q. (2020). Altered Brain Functional Connectivity Density in Fast-Ball Sports Athletes With Early Stage of Motor Training. *Frontiers in psychology, 11*, 530122. doi:10.3389/fpsyg.2020.530122

Zhang, K., Jan, Y.-K., Liu, Y., Zhao, T., Zhang, L., Liu, R., . . . Cao, C. (2022). Exercise intensity and brain plasticity: What’s the difference of brain structural and functional plasticity characteristics between elite aerobic and anaerobic athletes? *Frontiers in human neuroscience, 16*. doi:10.3389/fnhum.2022.757522

Zhou, W., Wu, J., Li, Y., Li, J., Sun, M., Li, R., . . . Gao, Q. (2023). The Antagonistic Alterations of Cerebellar Functional Segregation and Integration in Athletes with Fast Demands of Visual-Motor Coordination. *Cognitive Computation*. doi:10.1007/s12559-023-10150-7

Zhuang, X., Mishra, V., Nandy, R., Yang, Z., Sreenivasan, K., Bennett, L., . . . Cordes, D. (2020). Resting-State Static and Dynamic Functional Abnormalities in Active Professional Fighters With Repetitive Head Trauma and With Neuropsychological Impairments. *Frontiers in neurology, 11*, 602586. doi:10.3389/fneur.2020.602586

Zuidema, T. R., Hou, J., Kercher, K. A., Recht, G. O., Sweeney, S. H., Chenchaiah, N.,…Kawata, K. (2024). Cerebral Cortical Surface Structure and Neural Activation Pattern Among Adolescent Football Players. *JAMA Network Open*, *7*(2), e2354235. [doi:10.1001/jamanetworkopen.2023.54235](https://doi.org/10.1001/jamanetworkopen.2023.54235)

**Supplementary Material 3**

**Table 3. Studies included in the meta-analysis (n=18).**

| **Studies** | **Field** | **TR/**  **TE**  **(ms)** | **FA** | **Slices** | **Eyes**  **(o/c)** | **time** | **Slice**  **Timing corr.** | **Motion**  **corr.** | **Nuisance reg.** | **FWHM**  **(mm)** | **Norm.**  **template** | **Band**  **-pass**  **filt.** | **Statistical Analysis** |
| --- | --- | --- | --- | --- | --- | --- | --- | --- | --- | --- | --- | --- | --- |
|  |  |  |  |  |  |  |  |  |  |  |  |  |  |
| **Jin et al., 2024** | 3.0 T | 2000/30 | 90° | 33 | c | 480s | discarded first 10 time points | Max translation <2 mm, rotation <2° | 24-paras,  WM, CSF. | 6 | MNI | 0.01–0.08 Hz | FDR correction  (p < 0.05) |
| **Qi et al., 2024** | 3.0 T | 2000/30 | 90° | 43 | o | 480s | discarded first 10 time points | Max translation <2 mm, rotation <2° | WM, CSF, GSR, 24-paras. | 6 | MNI | 0.01–0.08 Hz | GRF correction (voxel p<0.001, cluster p<0.05) |
| **Yan et al., 2024** | 3.0 T | 2000 /30 | 90° | 75 | c | N/A | discarded first 10 time points | Max translation <2 mm, rotation <1° | 24-paras,  WM, CSF. | 6 | MNI | 0.01–0.08 Hz | GRF correction (voxel p<0.001, cluster p<0.05) |
| **Zhang, J.,**  **et al., 2024** | 3.0 T | 3000 /30 | 90° | 43 | N/A | N/A | discarded first 10 time points | Applied | N/A | 6 | N/A | 0.01–0.08 Hz | Alphasim correction |
| **Zhou et al., 2024** | 3.0 T | 2000 /30 | 90° | 58 | N/A | N/A | Applied | Applied | WM, CSF, GSR,  24-paras. | 4 | MNI | 0.01–0.08 Hz | FWE correction (voxel p<0.001, cluster p<0.05 size>50 voxels). |
| **de la Cruz et al., 2022** | 3.0 T | 484/30 | 90° | 56 | o | N/A | Applied | Applied | 12 paras, WM, RVT, ANATICOR,  RETROICOR | 6 | MNI | 0.01 - 0.1Hz | AFNI’s 3dClustSim, (voxel p<0.001, cluster p<0.05 size>59 voxels). |
| **Cao et al., 2020** | 3.0 T | 2000 / 30 | 90° | N/A | c | 480s | discarded first 10 time points | Applied | WM, CSF, head motion parameters | 4 | MNI | 0.01–0.08 Hz | TFCE (p<0.05) |
| **Ogino et al., 2021** | 3.0 T | 2500 / 30 | 80° | 40 | o | 375s | discarded first 4 time points | Applied | Applied | 8 | MNI | 0.01 - 0.1 Hz | FWE correction (voxel p<0.001, cluster p<0.05) |
| **Zhang et al., 2021** | 3.0 T | 2000 / 30 | 90° | 37 | c | 480s | discarded first 5 time points | Max translation <2 mm, rotation <2° | 6-paras. | 4 | MNI | 0.01–0.08 Hz | FWE correction (voxel p<0.01, cluster p<0.05) |
| **Shi et al., 2020** | 3.0 T | 2000 / 30 | 90° | 33 | c | 486s | discarded first 10 time points | Max translation <2 mm, rotation <2° | N/A | 8 | MNI | 0.01–0.08 Hz | GRF correction (voxel p<0.01, cluster p<0.05) |
| **Berti et al., 2019** | 1.5 T | 2500 / 40 | N/A | 23 | o | 480s | Discarded first 3 time points | Max translation <1 mm, rotation <1° | CompCor,  WM, CSF. | N/A | N/A | 0.01 - 0.1 Hz | FDR correction (p<0.05) |
| **Lu et al., 2018*** | 3.0 T | 2000 / 30 | 90° | 43 | o | 480s | discarded first 10 time points | Max translation <2 mm, rotation <2° | WM, CSF,  24-paras. | 4 | MNI T1 | 0.01–0.1Hz | GRF correction (voxel p< 0.001, cluster p<0.01) |
| **Liu et al., 2017** | 3.0 T | 2000/35 | 90° | 41 | c | 480s | discarded first 10 time points | Max translation <3 mm, rotation <3° | 6-paras. | 4 | MNI T1 | 0.01–0.08 Hz | Alphasim correction (voxel p<0.05, cluster p<0.05) |
| **Tan et al., 2017** | 3.0 T | 2000/35 | 90° | N/A | c | N/A | Applied | Applied | Applied | 6 | MNI | 0.01–0.08 Hz | AlphaSim correction (p<0.05) |
| **Kim et al., 2016** | 3.0 T | 3000 / 40 | 90° | 40 | N/A | 720s | Applied | Applied | WM, CSF. | 4 | NA | 0.01–0.08 Hz | FWE correction (p<0.05，size≥40 voxels). |
| **Raichlen et al., 2016** | 3.0 T | 2400 / 30 | 90° | 32 | o | 360s | discarded first 4 time points | Applied | aCompCor,  6 paras, WM, CSF. | 8 | Applied | 0.008 – 0.09 Hz | FDR correction (p<0.05) |
| **Kim et al., 2015** | 3.0 T | 3000 / 40 | 90° | 40 | c | 720s | Applied | Applied | WM, CSF, head motion covariates. | 4 | Applied | 0.01 – 0.08 Hz | FWE correction (p<0.05, size >40 voxels). |
| **Di et al., 2012** | 3.0 T | 2000 / 30 | 90° | 32 | o | 360s | discarded first 2 time points | Max translation <3 mm, rotation <3° | WM, CSF,  6-paras,  aCompCor. | 8 | MNI | 0.01 – 0.08 Hz | FDR correction (voxel p<0.001, cluster p<0.05 size>40 voxels). |

**Note: Abbreviations:** aCompCor: Anatomical Component-based Noise Correction; AlphaSim: Monte Carlo-based correction method; Band-pass filt: band-pass filtering; CSF: Cerebrospinal Fluid signal; Eyes (o/c):Eyes (open/closed); FA: Flip Angle; FDR correction: False Discovery Rate correction; FWE correction: Family-Wise Error correction; FWHM: Full Width at Half Maximum; GRF correction: Gaussian Random Field theory-based correction; MNI: Montreal Neurological Institute; Motion corr.: Motion correction; N/A: Not Available; Norm. template: Normalization template; Nuisance reg.: Nuisance regression; RETROICOR: Retrospective Image Correction for physiological noise; RVT: Respiration Volume per Time; Slice Timing corr.: Slice timing correction; TE: Echo Time; TFCE: Threshold-Free Cluster Enhancement; TR: Repetition Time; WM: White Matter signal; 6/24-paras: 6/24 motion parameters.

**Supplementary Material 4**

Checklist for inclusion of quality of literature: assessing the methodological quality of articles

**Table 4. Quality assessment of studies in meta-analysis.**

|  | Jin  et al.,  2024 | Qi  et al.,  2024 | Yan  et al.,  2024 | Zhang, J., et al., 2024 | Zhou  et al.,  2024 | de la  Cruz  et al.,  2022 | Ogino  et al.,  2021 | Zhang  et al.,  2021 | | Cao  et al.,  2020 | | | Shi  et al.,   2020 | | Berti  et al.,  2019 | | Lu  et al.,  2018 | Liu  et al.,  2017 | | Tan  et al.,  2017 | Kim  et al.,  2016 | Raichlen  et al.,  2016 | Kim  et al.,  2015 | Di  et al.  2012 |
| --- | --- | --- | --- | --- | --- | --- | --- | --- | --- | --- | --- | --- | --- | --- | --- | --- | --- | --- | --- | --- | --- | --- | --- | --- |
| Category 1: Sample characteristics (4) | | | | | | | | |  | | |  | |  | |  | | | | | | | | |
| 1. Important demographic data (age, gender, and training period) were reported with a mean (or median) and standard deviations (or range) (2) | 2 | 2 | 2 | 2 | 1 | 2 | 2 | | 2 | | 2 | | 2 | | 2 | 2 | | | 2 | 2 | 2 | 2 | 2 | 2 |
| 2. Control information (no experience with sports) (1) | 1 | 1 | 1 | 0 | 1 | 1 | 1 | | 1 | | 1 | | 1 | | 1 | 1 | | | 1 | 1 | 1 | 1 | 1 | 1 |
| 3. Sample size per group ≥ 10 (1) | 1 | 1 | 1 | 1 | 1 | 1 | 1 | | 1 | | 1 | | 1 | | 1 | 1 | | | 1 | 1 | 1 | 1 | 1 | 1 |
| Category 2: Methodology and reporting (8) | | | | | | | | |  | | |  | |  | |  | | | | | | | | |
| 1. All neuroanatomic measurements were taken without considering group assignment or subject identity (1) | 0 | 0 | 0 | 0 | 0 | 0 | 0 | | 0 | | 0 | | 0 | | 0 | 0 | | | 0 | 0 | 0 | 0 | 0 | 0 |
| 2. Magnet strength at least 1.5 T (1) | 1 | 1 | 1 | 1 | 1 | 1 | 1 | | 1 | | 1 | | 1 | | 1 | 1 | | | 1 | 1 | 1 | 1 | 1 | 1 |
| 3. MRI slice-thickness ≤ 3 mm and more than 1 slice was identified and traced (1) | 1 | 0 | 1 | 1 | 1 | 0 | 0 | | 1 | | 0 | | 1 | | 0 | 1 | | | 1 | 1 | 1 | 1 | 1 | 0 |
| 4. The acquisition and preprocessing techniques were clearly described so that they could be reproduced (1) | 1 | 1 | 1 | 0 | 0 | 0 | 0 | | 1 | | 0 | | 1 | | 1 | 1 | | | 1 | 1 | 0 | 0 | 0 | 1 |
| 5. Measurements were clearly described so that they could be reproduced (1) | 1 | 1 | 1 | 1 | 1 | 1 | 1 | | 1 | | 1 | | 1 | | 1 | 1 | | | 1 | 1 | 1 | 1 | 1 | 1 |
| 6. Coordinates were reported in a standard space unless there was no significant difference (1) | 1 | 1 | 1 | 1 | 1 | 1 | 1 | | 1 | | 1 | | 0 | | 1 | 1 | | | 1 | 1 | 1 | 1 | 1 | 1 |
| 7. Significant results are reported after correction for multiple testing using a standard statistical procedure (FDR, FWE, or permutation-based methods) (1) | 1 | 0 | 1 | 0 | 1 | 1 | 1 | | 1 | | 1 | | 0 | | 1 | 1 | | | 1 | 1 | 1 | 1 | 1 | 1 |
| 8. Conclusions were consistent with the results obtained and the limitation were discussed (1) | 1 | 1 | 1 | 1 | 1 | 1 | 1 | | 1 | | 1 | | 1 | | 1 | 1 | | | 1 | 1 | 1 | 1 | 1 | 1 |
| Total 12 | **11** | **10** | **11** | **8** | **9** | **9** | **9** | | **11** | | **9** | | **9** | | **10** | **11** | | | **11** | **11** | **10** | **10** | **10** | **10** |

**Table 5. Quality assessment of studies using seed-based functional connectivity approach in meta-analysis.**

|  | **Study selection** | | | | **Comparability** | | **Exposure** | | | **Statistical inference** | |
| --- | --- | --- | --- | --- | --- | --- | --- | --- | --- | --- | --- |
|  | Def | Repres | Sel ctrl | Def ctrl | Age & Gender | Other | Exp | Drop-out | Beh | Uncorrected p-value threshold | FP corrections |
| Jin et al., 2024 | + | + | + | + | - | - | + | - | ? | ? | + |
| Qi et al., 2024 | + | + | + | + | + | - | + | + | - | ? | - |
| Zhou et al., 2024 | + | + | + | + | + | + | + | + | + | + | + |
| Zhang, J., et al., 2024 | ? | + | - | + | + | + | + | - | - | - | - |
| Yan et al., 2024 | + | + | + | + | + | + | + | ? | ? | + | + |
| de la Cruz et al., 2022 | + | + | + | + | + | - | + | + | - | + | + |
| Ogino et al., 2021 | + | + | + | + | + | + | + | - | ? | + | + |
| Zhang et al., 2021 | + | + | + | - | ? | - | + | + | ? | - | + |
| Cao et al., 2020 | + | + | + | + | + | + | + | - | + | - | - |
| Shi et al., 2020 | + | + | + | + | + | - | + | ? | + | + | + |
| Berti et al., 2019 | + | + | + | + | ? | + | + | - | + | - | + |
| Lu et al., 2018 | + | + | + | + | + | + | + | + | - | + | - |
| Tan et al., 2017 | + | + | + | ? | + | - | + | + | - | - | - |
| Liu et al., 2017 | ? | + | + | - | + | - | + | + | - | - | - |
| Kim et al., 2016 | + | + | + | + | + | - | + | ? | ? | ? | + |
| Raichlen et al., 2016 | + | + | + | + | + | + | + | - | + | + | + |
| Kim et al., 2015 | ? | ? | ? | + | + | ? | + | - | ? | + | + |
| Di et al., 2012 | ? | ? | + | + | + | - |  | - | - | + | + |
| Kappa：**0.70** | 0.85 | 0.61 | 0.63 | 0.84 | 0.81 | 0.62 | 1 | 0.26 | 0.74 | 0.66 | 0.72 |

Note: The modified Newcastle-Ottawa Scale (NOS). Study-level risk of bias ratings and the kappa inter-rater agreement. For each item, we gave a score of “+”, if it was handled properly, “-”, if it did not meet the criteria at all, and “?”, if the information provided in the manuscript was unclear or missing. Abbreviations: Beh: behavioral; Clust: cluster forming threshold; Ctrl: control group; Def: definition; Diff: difference; Exp: exposure; FP: False-positives correction; Repres: representativeness.

**Supplementary Material 5**

**Table 6. Jackknife Sensitivity Analysis of meta-analysis results in FC.**

| All studies but | ALE | | | | SDM | | | | |
| --- | --- | --- | --- | --- | --- | --- | --- | --- | --- |
|  | **Increased FC** | | | | **Increased FC** | | | |  |
|  | **PCL** | **MeFG** | **Precuneus** | **IPL** | **DCG** | **SMG** | **RO** | **IFG** | **DCG** |
| Jin et al., 2024 | **√** | **√** | **√** | **√** | **√** | **√** | **×** | **√** | **√** |
| Zhou et al., 2024 | **×** | **×** | **×** | **√** | **√** | **√** | **√** | **√** | **-** |
| Zhang, J., et al., 2024 | **√** | **√** | **√** | **×** | **√** | **√** | **×** | **√** | **√** |
| Yan et al., 2024 | **√** | **√** | **√** | **√** | **√** | **√** | **√** | **√** | **√** |
| de la Cruz et al., 2022 | **√** | **√** | **√** | **×** | **√** | **√** | **√** | **×** | **√** |
| Ogino et al., 2021 | **√** | **√** | **√** | **√** | **√** | **×** | **×** | **×** | **-** |
| Zhang et al., 2021 | **√** | **√** | **√** | **√** | **√** | **√** | **√** | **√** | **-** |
| Cao et al., 2020 | **√** | **√** | **√** | **√** | **√** | **√** | **√** | **√** | **√** |
| Berti et al., 2019 | **√** | **√** | **√** | **√** | **√** | **√** | **√** | **√** | **-** |
| Lu et al., 2018 | **-** | **-** | **-** | **-** | **√** | **√** | **√** | **√** | **-** |
| Tan et al., 2017 | **√** | **√** | **√** | **√** | **√** | **√** | **√** | **√** | **√** |
| Liu et al., 2017 | **√** | **√** | **√** | **√** | **√** | **√** | **√** | **√** | **√** |
| Kim et al., 2016 | **√** | **√** | **√** | **√** | **√** | **√** | **√** | **√** | **√** |
| Raichlen et al., 2016 | **×** | **×** | **×** | **√** | **√** | **√** | **√** | **√** | **√** |
| Kim et al., 2015 | **√** | **√** | **√** | **√** | **√** | **√** | **√** | **√** | **√** |
| Total | **12/14** | **12/14** | **12/14** | **12/14** | **15/15** | **14/15** | **12/15** | **13/15** | **10/10** |

**Note:** Abbreviations: ALE: activation likelihood estimation; DCG: median cingulate / paracingulate gyri; FC: functional connectivity; IFG: inferior frontal gyrus; IPL: inferior parietal lobule; MeFG: medial frontal gyrus; SDM: seed-based d Mapping; SMG:supramarginal gyrus; PCL: paracentral lobule; RO: Rolandic operculum.

**Table 7. Jackknife Sensitivity Analysis of meta-analysis results**

**in ReHo, fALFF/ALFF.**

| All studies but | Metrics | ALE | | SDM |
| --- | --- | --- | --- | --- |
|  |  | **Increased ALFF, ReHo** | | **Increased ALFF, ReHo** |
|  |  | **PCG** | **LG** | **CB** |
| Jin et al., 2024 | ALFF | **√** | **√** | **×** |
| Qi et al., 2024 | fALFF | **√** | **√** | **√** |
| Zhang, J., et al., 2024 | m/zALFF  m/zfALFF | **√** | **√** | **√** |
| Zhang et al., 2021 | fALFF | **√** | **√** | **×** |
| Shi et al., 2020 | ALFF | **√** | **√** | **√** |
| Lu et al., 2018 | ALFF | **√** | **√** | **√** |
| Di et al., 2012 | ALFF | **√** | **√** | **×** |
| Yan et al., 2024 | ReHo | **×** | **×** | **√** |
| Zhang et al., 2024 | ReHo | **×** | **×** | **×** |
| Zhang et al., 2021 | ReHo | **×** | **×** | **√** |
| Total | | **7/10** | **7/10** | **6/10** |

**Note:** Abbreviations: ALE: activation likelihood estimation; ALFF: amplitude of low-frequency fluctuation; CB: Cerebellum; fALFF: fractional ALFF; LG: lingual gyrus; mALFF: mean ALFF; PCG: posterior cingulate gyrus; SDM: seed-based d Mapping; ReHo: regional homogeneity; zALFF: z-transformed ALFF.

**Table 8. Meta-regression analysis.**

| **Region** | | **MNI coordinate** | | | **SDM-z** | **p-value** | **Number of voxels** |
| --- | --- | --- | --- | --- | --- | --- | --- |
|  |  | **X** | **Y** | **Z** |  |  |  |
| **Effect of spatial smoothing kernel size in FC** | | | | | | |  |
|  | Right inferior frontal gyrus, opercular part, BA 44 | 54 | 16 | 16 | 2.811 | <0.05 | 106 |

**Note:** Abbreviations: BA, brodmann area; FC: functional connectivity; MNI, Montreal Neurological Institute; SDM, signed differential mapping; p < 0.05 (FWE), Cluster number ≥ 78 voxels.


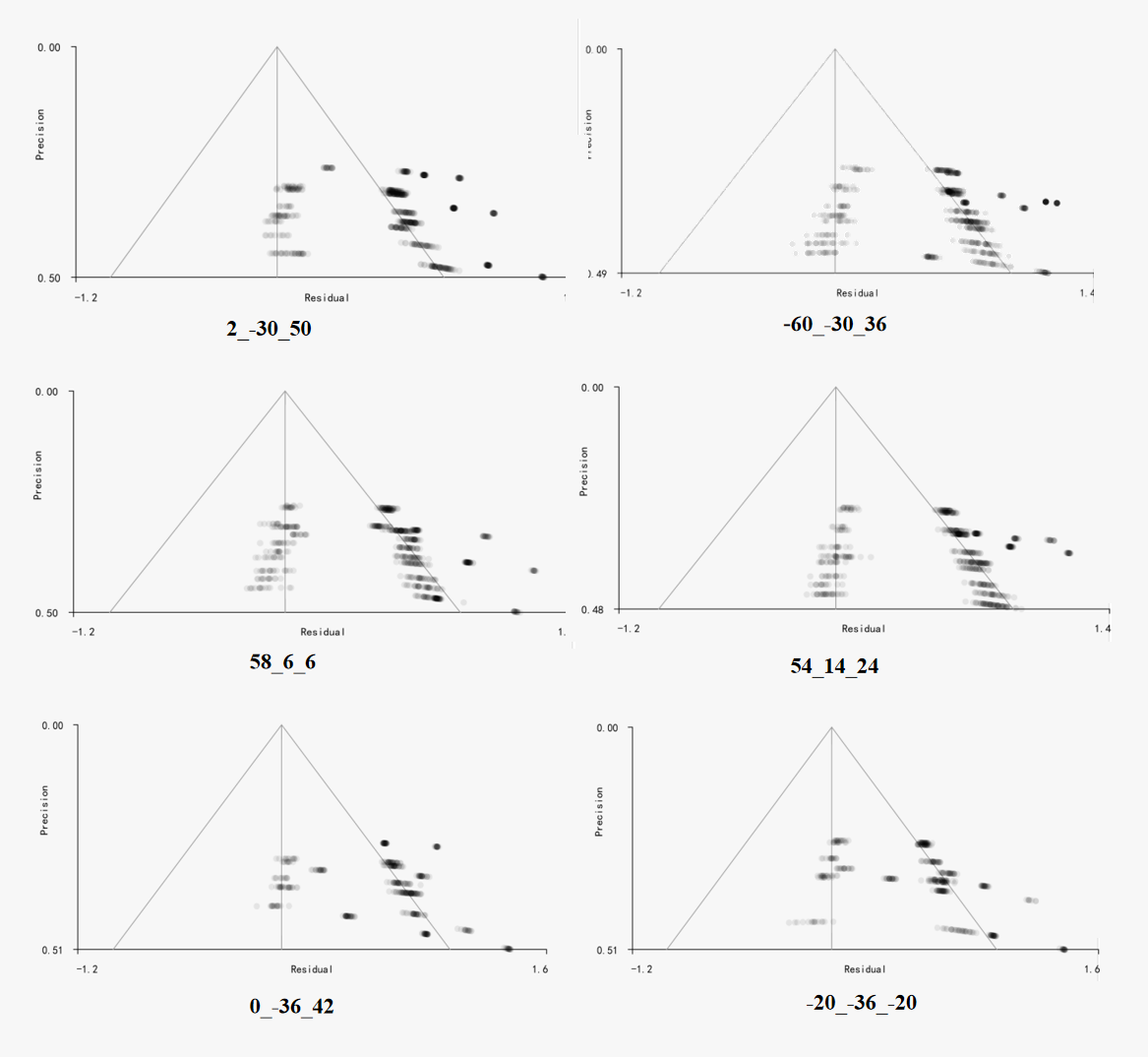


**Fig.1 Funnel plots for the main clusters revealed in the main SDM-PSI meta-analysis.**


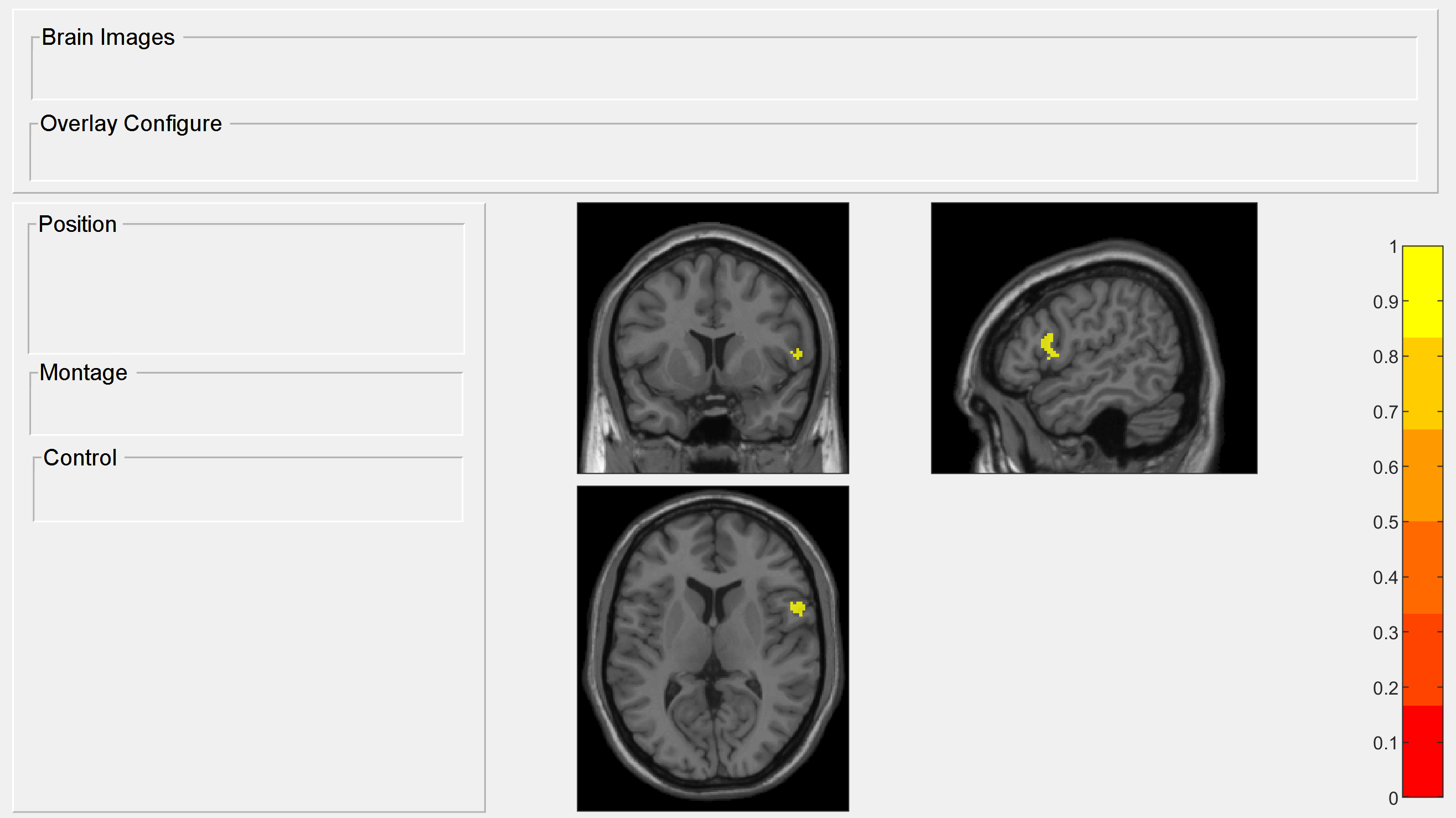

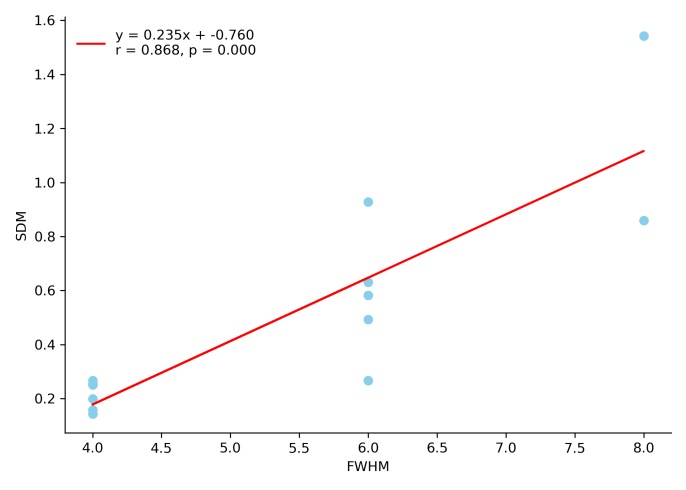


**Fig.2 Spatial smoothing kernel size are associated with right inferior frontal gyrus in FC meta-regression analysis.** No significant relationship was found between MRI acquisition parameters (e.g., TR, TE) and seed-based FC or regional functional activity changes.

**Supplementary Material 6**

**Method**

**Search strategy**

We conducted a systematic search of PubMed, Web of Science, Scopus (common databases) and MEDLINE, SPORTDiscus and PsycINFO (databases focusing on psychology, sports science and neuroimaging) from the initial emergence of resting-state research around 2010 until 2024.

The MeSH terms and free text content included in the search query.: (((((("Athletes"[Mesh Terms]) OR (sports)) OR (experts)) OR (profess*)) OR (players)) AND ((((("Magnetic Resonance Imaging"[Mesh Terms]) OR (functional magnetic resonance imaging)) OR (resting-state functional magnetic resonance imaging)) OR (resting-state)) OR (rs-fMRI)) AND ((((((((((((functional connectivity[Mesh Terms]) OR (connectivity)) OR (FC)) OR (rs-FC)) OR (amplitude of low-frequency fluctuation)) OR (ALFF)) OR (regional homogeneity)) OR (ReHo)) OR (degree centrality)) OR (DC)) OR (independent component analysis)) OR (ICA)) OR (functional connectivity destiny)). To ensure the inclusion of the most recent literature, we updated the literature searches every five months as follows:

**Phase I：**During the initial search on January 26, 2024, we included 23 systematic studies and 11 studies eligible for seed-based functional connectivity meta-analysis.

**PHASE II:** We conducted an updated search on June 9, 2024, using the same search strategy as before, which resulted in the addition of 4 systematic studies and 3 meta-analysis studies.

**PHASE III:** A third update of the literature search on November 13, 2024. We finally additional included 4 systematic studies and 1 study eligible for meta-analysis.

Ultimately, after integrating all the studies in these phases, a total of 31 studies were included in the systematic analysis and 15 studies were included in the meta-analysis study.

**Study exclusion and inclusion criteria**

The criteria for inclusion and exclusion are specified from four main perspectives: (1) subject selection, (2) methodology, (3) reporting of results, (4) article quality. In addition, we used the following two scales for assessment in article quality verification.

**1: Modified 12-point checklist**

The checklist consists of 8 questions. Included are questions on assessing participant characteristics (4 points) and methods and outcomes of imaging studies (8 points). This list is intended to be used only as an objective tool to assess the rigour of individual studies (Iwabuchi et al., 2015).

**2: Modiﬁed version of the Newcastle–Ottawascale (NOS)**

This modiﬁed version of the Newcastle–Ottawascale (NOS) , which is specific to fMRI data (Gentili et al., 2021), has 3 sections: Inclusion of studies (4 items); Comparability (2 items); Exposure (3 items); and Statistical inference (2 items). Two researchers rated the information about the included studies as Reliable (+); Uncertain (?); and unreliable (-). Subsequently, inter-rater agreement coefficients were calculated to determine the degree of risk bias of the results. Based on the scores were classified as 0-3 (high risk of bias), 4-7 (moderate risk of bias) and 7-11 (low risk of bias).

Two authors checked the inclusion of subjects and the methodological quality of each study, and subsequently, the reliability of the results was assessed by the risk bias assessment. The third investigator discussed the disagreement and decided.

Iwabuchi, S. J., Krishnadas, R., Li, C., Auer, D. P., Radua, J., & Palaniyappan, L. (2015). Localized connectivity in depression: A meta-analysis of resting state functional imaging studies. *Neuroscience & Biobehavioral Reviews*, *51*, 77–86. https://doi.org/10.1016/j.neubiorev.2015.01.006

Gentili, C., Benvenuti, S. M., Lettieri, G., Costa, C., & Cecchetti, L. (2018). ROI and phobias: The effect of ROI approach on an ALE meta‐analysis of specific phobias. *Human Brain Mapping,* *40*(6), 1814–1828. <https://doi.org/10.1002/hbm.24492>
